# Osmoregulation in freshwater anaerobic methane-oxidizing archaea under salt stress

**DOI:** 10.1093/ismejo/wrae137

**Published:** 2024-07-20

**Authors:** Maider J Echeveste Medrano, Andy O Leu, Martin Pabst, Yuemei Lin, Simon J McIlroy, Gene W Tyson, Jitske van Ede, Irene Sánchez-Andrea, Mike S M Jetten, Robert Jansen, Cornelia U Welte

**Affiliations:** Department of Microbiology, Radboud Institute for Biological and Environmental Sciences (RIBES), Radboud University, Heyendaalseweg 135, 6525AJ Nijmegen, The Netherlands; Centre for Microbiome Research (CMR), School of Biomedical Sciences, Queensland University of Technology (QUT), Translational Research Institute (TRI), 37 Kent Street, Woolloongabba, QLD 4102, Australia; Department of Environmental Biotechnology, TU-Delft University, Van der Maasweg 9, 2629HZ Delft, The Netherlands; Department of Environmental Biotechnology, TU-Delft University, Van der Maasweg 9, 2629HZ Delft, The Netherlands; Centre for Microbiome Research (CMR), School of Biomedical Sciences, Queensland University of Technology (QUT), Translational Research Institute (TRI), 37 Kent Street, Woolloongabba, QLD 4102, Australia; Centre for Microbiome Research (CMR), School of Biomedical Sciences, Queensland University of Technology (QUT), Translational Research Institute (TRI), 37 Kent Street, Woolloongabba, QLD 4102, Australia; Department of Environmental Biotechnology, TU-Delft University, Van der Maasweg 9, 2629HZ Delft, The Netherlands; Department of Environmental Sciences for Sustainability, IE University, C. Cardenal Zúñiga 12, 40003 Segovia, Spain; Laboratory of Microbiology, Wageningen University, Stippeneng 4, 6708WE Wageningen, The Netherlands; Department of Microbiology, Radboud Institute for Biological and Environmental Sciences (RIBES), Radboud University, Heyendaalseweg 135, 6525AJ Nijmegen, The Netherlands; Department of Microbiology, Radboud Institute for Biological and Environmental Sciences (RIBES), Radboud University, Heyendaalseweg 135, 6525AJ Nijmegen, The Netherlands; Department of Microbiology, Radboud Institute for Biological and Environmental Sciences (RIBES), Radboud University, Heyendaalseweg 135, 6525AJ Nijmegen, The Netherlands

**Keywords:** compatible solutes, methanotroph, salinity adaptation, ANME, metabolomics, “Ca. Methanoperedens”

## Abstract

Climate change–driven sea level rise threatens freshwater ecosystems and elicits salinity stress in microbiomes. Methane emissions in these systems are largely mitigated by methane-oxidizing microorganisms. Here, we characterized the physiological and metabolic response of freshwater methanotrophic archaea to salt stress. In our microcosm experiments, inhibition of methanotrophic archaea started at 1%. However, during gradual increase of salt up to 3% in a reactor over 12 weeks, the culture continued to oxidize methane. Using gene expression profiles and metabolomics, we identified a pathway for salt-stress response that produces the osmolyte of anaerobic methanotrophic archaea: N(ε)-acetyl-β-L-lysine. An extensive phylogenomic analysis on N(ε)-acetyl-β-L-lysine-producing enzymes revealed that they are widespread across both bacteria and archaea, indicating a potential horizontal gene transfer and a link to BORG extrachromosomal elements. Physicochemical analysis of bioreactor biomass further indicated the presence of sialic acids and the consumption of intracellular polyhydroxyalkanoates in anaerobic methanotrophs during salt stress.

## Introduction

Coastal freshwater wetlands are dynamic ecosystems and hotspots for methane cycling, a greenhouse gas of growing concern [1, 2]. Biological methane production in these systems is performed by marine methanogenic archaea present in deeper sediment layers [3]. Methanotrophs constitute a natural methane biofilter that mitigates part of the coastal methane emissions [4, 5]. In the deeper anoxic layers of the sediment, anaerobic methanotrophic archaea (ANME) couple methane oxidation to the reduction of a range of electron acceptors including nitrate, sulfate, metal oxides, and natural organic matter [4, 6–8]. In cases where nitrate is the most competitive electron acceptor, members of the archaeal family *Methanoperedenaceae* (formerly ANME-2d) are thought to dominate the removal of methane [5]. In the top sediment layer and water column of coastal systems, methane oxidation occurs mostly under oxic conditions by methanotrophic bacteria [9, 10].

Coastal freshwater regions are currently facing a plethora of anthropogenic challenges, including intensive agricultural land use and climate change–driven sea level rise [11, 12]. Sea water intrusion to coastal ecosystems poses hyperosmotic pressure on freshwater microbial communities, including bacterial methanotrophic communities [13, 14]. This osmotic pressure makes the *Methanoperedenaceae* ANME a relevant group of microorganisms to investigate regarding their stress response to salt concentration increase; salinity-derived methane emission reduction has been described for aquatic inland waters [15], but the effect on anaerobic methanotrophs has to be further unraveled. Investigations on osmoregulation are strongly biased toward bacteria or halophilic archaea with a few exceptions in methanogens such as *Methanosarcina mazei* [16–18], thereby leaving the comprehensive physiological response of ANME to salt stress largely unexplored [19].

One of the most common microbial osmoregulation strategies consists of intracellular osmolyte accumulation. Osmolytes are water-soluble organic compounds that accumulate intracellularly to counter hyperosmotic pressure [17, 20–22]. Osmolytes are categorized into sugars, amino acids, polyols, and their derivatives. A special case are polyhydroxyalkanoates (PHAs), well-described storage polymers, that confer cell stability to halophilic microorganisms [23, 24]. In this vein, “*Candidatus* Methanoperedens nitroreducens” has been reported to accumulate PHAs [25–27].

Protein glycosylation has been found to be another possible strategy to adapt to saline stress conditions. For example, posttranslational protein modifications are used as a response to changes in salinity, where, for example, the S-layer glycoprotein of *Haloferax volcanii* was found to show a distinct glycan decoration for either cells grown at high (3.4 M NaCl) or low (1.75 M NaCl) salt concentrations [28]. Furthermore, sialic acids, negatively charged monosaccharides that can modify surface layer proteins, flagella, or other extracellular proteins such as extracellular polymeric substances (EPS), are yet another potential salinity-stress-related adaptation in microorganisms [29–33]. Along these lines, de Graaff *et al.* presented evidence that sialic acids are found on the EPS of environmental bacteria in sea water–adapted aerobic granular sludge [34].

Here, we evaluated the metabolic and physiological response of a bioreactor enriched in “*Ca.* Methanoperedens” ANME to salinity stress. A complementary array of methods including bioreactor enrichment, methane oxidation activity assays, PHA visualization and quantification, and fluorescent *in situ* hybridization (FISH) microscopy as well as metagenomics, metatranscriptomics, metaproteomics, and nontargeted metabolomics, revealed that *“Ca.* Methanoperedens” produces the osmolyte N(ε)-acetyl-β-L-lysine. We revealed that the responsible genes were probably horizontally acquired from bacterial taxa and can be also encoded on extrachromosomal elements termed BORGs [35]. Our investigation also shed light on the overall stress response, transcriptionally detected morphology change, storage polymer usage, and EPS production by freshwater “*Ca.* Methanoperedens” archaea.

## Materials and methods

### “*Ca.* Methanoperedens” microcosm experiment at increasing salinities

Prior to the bioreactor experiment, we first performed a preliminary 18-day-long salinity stress microcosm experiment on the same “*Ca.* Methanoperedens” enrichment. Here, we determined the methane oxidation potential of the enrichment at three different salinities: 1%, 2%, and 3%. Freshwater and sulfate-free ASW medium had otherwise the same composition as specified for the bioreactor experiment. For the microcosm experiment, pH was adjusted with NaOH pellets to 7.3 and buffered with 10 mM HEPES to secure pH values between 7.2 and 7.5. Prior to inoculation with bioreactor material, medium was made anoxic by Ar:CO_2_ (95:5) sparging for 2 h. A total volume of 30 ml was sampled per biological replicate and immediately transferred to the anaerobic chamber in capped and anoxic 60 ml syringes. To remove precipitates, biomass was washed three times with the respective medium. Briefly, medium was dispensed in final volumes of 20 ml in 120 ml serum bottles. Overpressure (Ar:CO_2_, 95:5) was checked to be around 1.3 bar in all bottles. Serum bottles were capped with red butyl rubber stoppers that were previously boiled twice in 100 mM NaOH and twice washed in water. Aluminum crimps were used to ensure full attachment of the stoppers. Later, biomass samples were subjected to an extra 1 min Ar:CO_2_ (95:5) sparging to ensure full anoxic conditions. Serum bottles then received 10% ^13^C-CH_4_ and 2 mM NaNO_3_. ^12^C-CO_2_ and labeled ^13^C-CO_2_ were measured in 50 μl headspace samples by gas chromatography–mass spectrometry (GC-MS), Agilent 8890 GC System, and Agilent 5977B GC/MSD (Agilent Technologies, Santa Clara, CA, USA). Calibration was performed with standard gas consisting of He/O_2_/N_2_/CH_4_/CO_2_/N_2_O with terminal values of (%): balance/1.02/1.03/1.05/1.04/0.050 (Linde Gas Benelux BV, Schiedam, The Netherlands). Nitrate consumption was followed by MQuantTM colorimetric test strips (Merck, Darmstadt, Germany). Overpressure and a stable pH (7.2–7.5) were monitored before every injection. Chromatography data were analyzed using the Agilent OpenLab CDS Software.

### Bioreactor setup and operation

A 2 L bioreactor (Applikon, Delft, The Netherlands) was anoxically inoculated with 1 L biomass of a “*Ca.* Methanoperedens Vercelli Strain 1” enrichment originating from freshwater Italian rice paddy fields [36].

The bioreactor experiment was run for 12 weeks. It was operated as a sequencing fed-batch reactor (SBR) resulting in granular biomass; the sequences consisted of 22 h 45 min of receiving medium (250–300 ml/day), 15 min of settling, and 45 min of supernatant removal, with ~4 days hydraulic retention time (Supplementary Fig. 1B). The bioreactor contained two standard six-blade turbines at 96 rpm and was operated at room temperature. The pH was buffered with 100 g/l KHCO_3_ solution and controlled by a BL 931700 pH controller Black Stone (Hanna Instruments, Rhode Island, USA) (Supplementary Fig. 1B). The bioreactor was constantly fed with CH_4_ with a flow of 10 ml/min and sparged with Ar:CO_2_ (95/5). Sulfate-free artificial sea water (ASW) [37] was increased stepwise by 0.25% every 2 weeks until 1.5% (brackish) was reached at 12 weeks (Supplementary Fig. 1A). To further investigate potential biomass acclimation, the bioreactor ran at 1.5% for over 2.5 months prior to increasing salinity to 3% (marine) in 0.5% steps over the course of a month (Supplementary Fig. 1A). The ASW composition was adapted and excluded MgSO_4_ to avoid growth of sulfate-reducing bacteria [37]. Traces of potassium, chlorides, and sulfate from the medium—together with sodium from the NaNO_3_ substrate—contributed between 0.01% and 0.1% to the baseline salinity, increasing or decreasing, depending on the nitrate demand (with an average 0.6 mmol NaNO_3_/day).

### Physicochemical analysis

Liquid nitrate consumption and/or nitrite toxicity were controlled daily in the bioreactor via MQuantTM colorimetric test strips (Merck, Darmstadt, Germany). From 0% to 1.5% salinity increase, ammonium was followed biweekly whereas during the 1-month 1.5%–3% salinity increase, it was done weekly. For this analysis, ammonium was determined either using a low- or high-sensitivity protocol (range from 40 to 400 μM or from 0.5 to 5 mM) after reaction with 10% orthophthaldialdehyde as previously described [38]. For the low-sensitivity assay, fluorescence was read with the Spark 10 M Plate Reader (Tecan, Grodig, Austria) whereas for the high-ammonium-sensitivity assay, samples were read at 420 nm absorbance with a spectrophotometer Spectra Max 190 (VWR, Boxmeer, The Netherlands).

### Bioreactor batch activity assays

To determine the methane oxidation rate of the bioreactor microbial community, the bioreactor was run as batch with 20% ^13^C-CH_4_ and additional N_2_ in the headspace to obtain an overpressure of 1.2–1.3 bar. The headspace was first flushed for 2–3 h with Ar:CO_2_ (95/5) to remove residual methane traces. The starting nitrate concentration was ~1.5 mM. Over a period of 4–5 days, labeled ^13^C-CO_2_ and ^12^C-CO_2_ were measured and nitrate limitation, overpressure, and a stable pH were controlled. Fifty microliters of bioreactor headspace samples were analyzed as described above. As normalizers for activity assays, dry weight (*n =* 2, 15 ml) and percentages of archaeal and bacterial 16S rRNA gene copy numbers (quantitative PCR, qPCR) were used. Our biomass grew in the form of granules and, for this growth type, dry weight was preferred over archaeal cell counts. This way, the variation derived from granule heterogeneity in between conditions was minimized.

### Nucleic acid extractions and cDNA synthesis

DNA for in-depth metagenomics was extracted at 0% and 1.5% salinity (*n =* 1). We added a third DNA sample at 3% salinity for a less deep (2 Gbp/sample) metagenome sequencing (*n =* 1). To obtain a detailed look at the microbial community shift via 16S rRNA gene amplicon analysis of bacteria and archaea, DNA was extracted biweekly. Additional DNA extractions for bacterial and archaeal abundance estimations via qPCR were employed at (in %): 0,0.75,1.5,1.5-acclimated, and 3. RNA was extracted at 0% and 1.5% salinities (in four biological replicates, *n* = 4). With the aim of achieving a targeted gene expression profile through reverse transcriptase qPCR, additional RNA extractions were performed at different salinities (in %): 0, 0.5, 1,1.5,1.5-acclimated, 3 (in three biological replicates per condition, *n =* 3).

DNA extractions were performed from 2 ml biomass using the DNeasy PowerSoil Kit (Qiagen, Hilden, Germany) and eluted DNA was stored at −20°C. Two-milliliter biomass for RNA extractions were anoxically sampled with four biological replicates (*n* = 4) per time point, freeze dried overnight and stored at −70°C. RNA was extracted with the RNeasy PowerSoil Kit (Qiagen, Hilden, Germany), with an initial manual pottering of samples to disrupt the granules. RNA extractions were stored at −70°C. Metatranscriptomics-directed RNA samples were DNAase I-treated at 37°C for 30 min with the solutions included in the RiboPure Bacteria Kit (Thermo Fisher Scientific, Waltham, MA, United States). For RNA samples used for RT-qPCR, we employed the same DNAase-treatment as the one suggested in the reverse transcriptase (RT) cDNA synthesis kit - the Revert Aid H Minus First Strand cDNA Synthesis Kit (Thermo Fisher Scientific, Waltham, MA, United States). DNA and RNA quality were determined using a NanoDrop Spectrophotometer ND-1000 (Isogen Life Science, Utrecht, Netherlands) and a Bioanalyzer 2100 (Agilent, Santa Clara, CA, United States), respectively. Concentrations were measured with a Qubit 2.0 fluorometer using the DNA dsDNA HS for DNA and RNA HS kit for RNA (Thermo Fisher Scientific, Waltham, MA, United States). Sequencing was performed by Macrogen Europe BV (Amsterdam, The Netherlands).

### Bacterial and archaeal 16S rRNA gene amplicon analysis

Gene amplicon sequencing was performed on a MiSeq (Illumina) platform, using library kit Herculase II Fusion DNA Polymerase NEXTERA XT Index kit V2 (Illumina, Eindhoven, Netherlands), generating 2 × 300 bp paired-end reads. See Supplementary Table 1 for bacterial and archaeal primers employed. Recovered 16S rRNA gene amplicon raw sequences were processed in R Studio version 2022.12.0 + 353 and R v4.2.2 with: DADA2 package v1.26.0, ggplot2 v3.4.0, phyloseq v1.42.0, vegan v2.6.4, DESEQ2 1.38.2, dendextend v1.16.2, tidyr v1.2.1, viridis v0.6.2, reshape v0.8.9, zoo v1.8.11 and plyr v1.8.8. First, we filtered out all flanking adapters attached to our primers with cutadapt v1.18 [39]. Briefly, we used the DADA2 pipeline [40] quality plots to trim forward and reverse bacterial reads at 270 bp and 200 bp whereas archaeal reads at 280 bp and 200 bp, respectively. Trimming was combined with trimLeft option 20 for primer removal. After error models were generated, sequences were dereplicated, merged and chimeras were discarded producing between 48 000–62 000 and 90 000–120 000 paired-end nonchimeric merged bacterial or archaeal sequences, respectively. Later, amplicon sequencing variants (ASVs) were inferred and assigned using the Silva database release v.138.1 [41]. ASVs were clustered by taxonomy and relative abundance using the R package phyloseq [42] and plotted with ggplot2 v3.4.0 [43].

### Quantitative PCR

For the determination of gene abundances in DNA samples, all qPCR amplifications consisted of 12 μl SYBR Green FastMix (QuantaBio, Beverly, MA, United States), 0.6 μl of forward and reverse primer (both at 10 μM) topped up with DEPC-treated water (Thermo Fisher Scientific, Waltham, MA, United States) yielding a total volume of 25 μl. See Supplementary Table 1 for bacterial and archaeal primer set employed. The qPCR program consisted of the following steps: initial denaturation (94°C for 5 min), denaturation (94°C for 30 s), annealing temperature (varying-T-°C for 30 s), elongation (72°C for 30 s), and melting curve (50–95°C, 0.5°C increase per 5 s). Denaturation, annealing, and elongation steps were repeated for 40 cycles.

For the gene expression determination of genes encoding for osmolyte production enzymes, qPCR primer sets specific for the *kamA* and *ablB* genes of “*Ca.* Methanoperedens” were synthesized (Biolegio, Nijmegen, The Netherlands; primer sequences and optimized annealing temperature in Supplementary Table 1). In brief, synthesized cDNA was used for quantitative PCR with a C1000 Touch thermocycler coupled with a CFX96 Touch Real-Time PCR detection system (Bio-Rad Laboratories, the Netherlands) with the same PCR mixtures and program reported Dalcin Martins *et al.* For comparison of gene expression, we calculated ΔΔCt values between the genes of interest and the 16S rRNA gene copy abundance of archaea. PCR efficiencies were obtained based on amplification slopes, keeping only amplifications with efficiencies above 90%. Single-copy bacterial or archaeal 16S rRNA gene-carrying pGEM-T Easy vector plasmids (with the same mentioned primer set) (Promega, The Netherlands) were used for quantification calibration.

### Metagenomics

Metagenomic sequencing was performed with a TruSeq DNA PCR free library using an insert size of 350 bp on a NovaSeq6000 (Illumina) platform, producing 2 × 151 bp paired-end reads (10Gbp/sample). Read quality was assessed with FASTQC v0.11.9 before and after quality trimming, adapter removal, and contaminant filtering, performed with BBDuk (BBTools v38.75). We employed Kaiju v1.7.2 for read-based taxonomical classification of trimmed reads, which also included the sample from 3% salinity [44]. Trimmed reads were co-assembled de novo using metaSPAdes v3.14.1 [45] and mapped to assembled contigs using BBMap (BBTools v38.75) [46]. Contigs at least 1000 bp long were used as template for read mapping of metatranscriptomic and metaproteomic sequences, as well as binning. Sequence mapping files were handled and converted using Samtools v1.10., later used for binning with CONCOCT v2.1 [47], MaxBin2 v2.2.7 [48], and MetaBAT2 v2.12.1 [49]. Resulting metagenome-assembled genomes (MAGs) were dereplicated with DAS Tool v1.1.1 [50] and taxonomically classified with the Genome Taxonomy Database Toolkit GTDB-Tk v2.1.0 [51]. For a metagenomic binning read-recruitment assessment together with a read-based “*Ca.* Methanoperedens” strains differentiation, SingleM v2 (https://github.com/wwood/singlem) was employed. MAG completeness and contamination was estimated with CheckM v1.1.3 [52]. Metagenome-assembled genomes were annotated with DRAM v1.0 [53] with default options, except min_contig_size at 1000 bp, and genes of interest were searched in annotation files as well as via BLASTp and HMM analyses. To corroborate poorly annotated genes/proteins, we opted to validate manual curations with the NCBI Batch Entrez Conserved Domains search option. See below for in-depth explanation for *kamA* and *ablB* gene mining and amino acid tree generation together with “*Ca*. Methanoperedens Strain Vercelli 1” biogeography study and additional metagenomic analysis, including: operon retrieval and sialic acid gene coverage calculations.

To obtain a read-based “*Ca.* Methanoperedens” presence *proxi* at 3% salinities an additional metagenome was generated. Library preparation of the metagenome was done using the Nextera XT kit (Illumina, San Diego, California U.S.A.) according to the manufacturer’s instructions. Enzymatical tagmentation was performed starting with 1 ng of DNA, followed by incorporation of the indexed adapters and amplification of the library. After purification of the amplified library using AMPure XP beads (Beckman Coulter, Indianapolis, USA), libraries were checked for quality and size distribution using the Agilent 2100 Bioanalyzer and the High sensitivity DNA kit. Quantitation of the library was performed by Qubit using the Qubit dsDNA HS Assay Kit (Thermo Fisher Scientific Inc Waltham USA). The libraries were pooled, denatured and sequenced with the MiSeq (Illumina) sequencer (San Diego, CA, USA). Paired end sequencing of 2 × 300 bp was performed using the MiSeq Reagent Kit v3 (San Diego, CA, USA) according to the manufacturer’s protocol yielding 4 584 058 reads.

### Biogeography on “Candidatus Methanoperedens Strain Vercelli 1”

The biogeography of “*Ca.* Methanoperedens Vercelli Strain 1,” identified by GTDB-Tk v2.1.0 as s__905339155.1, was assessed by querying operational taxonomic units (OTUs) categorized under the same species. This search utilized keywords for species numbers and was conducted across publicly available metagenomes using Sandpiper (July 2022) (https://sandpiper.qut.edu.au/) (see Supplementary Table 2).

### Gene-based metagenomic analysis

Softwares Artemis v16.0.0 and Operon-mapper (https://biocomputo.ibt.unam.mx/operon_mapper/) (accessed on August 16, 2023) were used for a comprehensive gene visualization and prediction of the operon conforming the osmolyte production genes of interest, respectively [54, 55]. Genes encoding sialic acid biosynthesis were obtained via BLASTp from reference pathways of *Campylobacter jejunii* [56] and *Halorubrum* sp. PV6 [57] with e-value thresholds below e^-20 including a protein identity threshold of >30%. To retrieve a read and contig size corrected gene coverage, we employed CoverM with the -contig flag, with a minimum identity of 95% and a minimum aligned read length of 75% for each read (https://github.com/wwood/CoverM). We also corroborated the annotation of genes involved in sialic acid biosynthesis with HMM profiles building PFAM annotations of genes of interest, with a hmm alignment threshold of 10e^-5. When possible, HMMs that were stringently KEGG-curated (K15897/*pseG* and K15912/*legB*) with predefined adaptive thresholds of kofamKOALA (https://www.genome.jp/tools/kofamkoala/) (accessed on September 2023) [58]. Both for PFAM and HMM annotations, relative abundances were calculated based on summed coverage per metagenome, either freshwater or brackish.

### Lysine 2,3-aminomutase and β-lysine N6-acetyl transferase phylogenetic trees

To expand the potential of N(ε)-acetyl-β-L-lysine biosynthesis across bacterial and archaeal representatives, we first obtained a list of genomes from the GTDB [58]. Using this list, we retrieved the corresponding genomes from the NCBI database. We then searched these genomes for the *kamA* (KEGG: K01843) and *ablB* (KEGG: K21935) genes, which are responsible for encoding the relevant amino acid sequences. This approach allowed us to identify and analyze the presence of these genes in the selected bacterial and archaeal genomes. “*Ca.* Methanoperedens”-related *kamA* and *ablB* sequences encoded on BORGs were directly downloaded from the reference paper [35]. The Al Shayeb *et al.* metagenome was screened for osmolyte biosynthesis genes of interest via HMMs that were stringently KEGG-curated with the default parameters of kofamKOALA [58]. Sequences were first aligned with MUSCLE v3.8.31 [59], trimmed with (−--gappy out) using Trimal v1.4.rev15 [60] and ran with IQ-TREE v2.0.3 using flags -st AA -m MFP -bb (ultrafast bootstrap) 1000 nt AUTO (model selected according to the Bayesian information criterion (BIC): LG + R10 both for *kamA*-encoded LAM and for *ablB*-encoded AT) [61]. The aligned *kamA*-encoding LAM sequences were categorized in two groups based on whether LAM was found alone (15/50) or with *ablB* on the same BORG element (14/50) (Supplementary Table 3). For the *ablB-*encoding AT tree, we only kept 14 sequences (out of the 37 mined) containing contiguous *kamA* (encoding LAM) and *ablB* (encoding AT) genes. In fact, both genes are regulated together (same operon) in order to produce N(ε)-acetyl-β-L-lysine (Supplementary Table 4). For certain “*Ca.* Methanoperdens” MAGs of interest, NCBI biosample was used to retrieve sampling source information, infer whether they were described to harbor a plasmid [62] or recover metatranscriptomic evidence of osmolyte gene expression following the same metatranscriptomic pipeline as described for this paper. We employed iTOL v5 for the annotation of our tree [63].

### Metatranscriptomics

Metatranscriptomic sequencing was performed using a TruSeq stranded mRNA library Kit (Illumina, San Diego, CA, United States) on a NovaSeq 6000 (Illumina) platform, generating 100-bp paired-end reads with 10 Gb throughput/sample. Raw sequences were quality trimmed using sickle v1.33 (https://github.com/najoshi/sickle) and ribosomal RNA contaminant-filtered, mapped against the DRAM-generated scaffolds and transcripts per million (TPM) values generated using trancriptm v0.2 (https://github.com/sternp/transcriptm). To visualize an annotated or categorized gene expression profile, we employed the R package ggpubr. For “*Ca.* Methanoperedens” specific individual gene expression contribution, new TPM values were calculated only including “*Ca.* Methanoperedens” contigs.

### Metaproteomics

Metaproteomic analysis was performed as recently described [33, 64]. For protein extraction and proteolytic digestion, ~100 mg of each cell pellet (wet weight) was dissolved in 175 μl 50 mM TEAB buffer (with 1% NaDOC) and 175 μl B-PER buffer (Thermo Scientific, Germany) by vortexing. Then, acid-washed glass beads (105–212 μm, Sigma-Aldrich) were added and the mixtures were vortexed thoroughly. Thereafter, the samples were spun down and the supernatant was collected and proteins were precipitated by adding 1 volume TCA to 4 volumes supernatant. The obtained protein pellets were once washed with ice cold acetone and then dissolved in 6 M urea (in 100 mM ammonium bicarbonate, ABC). Further, the disulfide bridges were reduced by the addition of 10 mM DTT and reduced using 20 mM IAA. 200 mM ABC buffer was then added to the samples to obtain a solution with <1 M urea. Finally, proteolytic digestion was performed by adding trypsin (0.1 μg/μl in 1 mM HCl, Sequencing Grade Modified Trypsin, Promega) at a ratio of 50:1 (w:w, protein:trypsin) to the sample. The proteolytic digestion was performed overnight at 37°C, under gentle shaking at 300 rpm. Peptides were desalted using an OASIS HLB solid phase extraction well plate (Waters, UK) according to the instructions of the manufacturer, speed vac dried and stored at −20°C until further processed. Shotgun proteomic analysis followed with ~500 ng of proteolytic digest and analysed using an EASY nano-LC 1200, equipped with an Acclaim PepMap RSLC RP C18 separation column (50 μm x 150 mm, 2 μm), and a QE plus Orbitrap mass spectrometer (Thermo Fisher Scientific, Germany). The flow rate was maintained at 350 nl/min over a linear gradient from 5% to 25% solvent B over 88 minutes, and finally to 55% B over 60 min. Data were acquired from 0 to 175 min. Solvent A was H_2_O containing 0.1% formic acid, and solvent B consisted of 80% ACN in H_2_O and 0.1% formic acid. The mass spectrometer was operated in data-dependent acquisition mode, where the top 10 most intense precursor ions were selected for fragmentation using higher-energy collisional dissociation (HCD) using a normalized collision energy (NCE) of 28. MS1 spectra were acquired from 385–1250 m/z at 70 K resolution, and an AGC target of 3e6, and a max IT of 75 ms. Fragmentation spectra were measured at 17 K resolution, with an isolation window of 2 m/z, an AGC target of 2e5, and a max IT of 75 ms. Unassigned, singly, and >5× charged ions were excluded from fragmentation. Mass spectrometric raw data were database searched using a protein reference database containing ORFs identified from the contigs of the metagenomics sequencing data obtained from the same community, using PEAKS Studio X (Bioinformatics Solutions Inc., Waterloo, Canada). Database searching was performed allowing 20 ppm parent ion and 0.02 m/z fragment ion mass error, 3 missed cleavages, carbamidomethylation as fixed and methionine oxidation and N/Q deamidation as variable modifications. Peptide spectrum matches were filtered for 1% false discovery rates (FDRs) and identifications with more than or equal to two unique peptides were considered as significant. Quantitative analysis of the changes between conditions, was performed using the PEAKSQ module (Bioinformatics Solutions Inc., Canada). Normalization was based on the total ion current (TIC), and only proteins with at least two unique peptides and identified in at least two out of three biological replicates were considered. Peptide spectrum matches were filtered with a 1% FDR. ANOVA was used to determine the statistical significance of the changes between the conditions. Proteomics raw data, reference sequence database, and database search files have been deposited in the ProteomeXchange consortium database with the dataset identifier PXD048239.

### Nontargeted metabolomics

Biomass from 5 ml samples (*n =* 4 per salinity) was collected in Nylon membrane filters (0.45 μm, 47 mm (WHA7404004) (Whatman, Maidstone, UK) using a vacuum system and were washed using ~5–10 ml of ice-cooled nitrate-free freshwater bioreactor medium. From here onward, samples were processed as described before [65], with some modifications. Washed biomass was vacuum-filtered using the same filter with acetonitrile:methanol:deionized water (40:40:20) (extraction solvent). Immediately after, filters were placed upside down and incubated for 10 min in 1.5 ml of the same extraction solvent in petri dishes that were standing on dry ice. To enhance cell disruption, we pipetted the extraction solvent ~20–25 times before transferring it to 1.5 ml tubes. Cell debris and proteins were removed by centrifugation for 10 min at 4°C and 20 000x*g*. Samples were collected and stored at −70°C until analysis on an Agilent 6546 Liquid Chromatography–Quadrupole Time of Flight (LC/Q-TOF) system, as specified previously [66]. In brief, samples were separated using aqueous normal phase chromatography, followed by MS detection in the positive ionization mode (m/z 50–1200). Vendor-specific datafiles were converted to mzXML format using MSconvert [67] and analyzed using XCMS online [68]. To filter for osmolytes, we selected for features with peak areas above half a million counts that increased with higher salinities. The unknown metabolite with m/z 189.125 was selected for MS2 fragmentation (unit isolation) and fragmented at collision energies of 10, 20, and 40 V. With the collected MS2 spectra, *in silico* structure prediction was performed using SIRIUS [69].

### Reference N(ε)-acetyl-β-L-lysine production with *M. mazei* and negative control

Reference osmolyte-producing *M. mazei* DSMZ 7222 was grown in 23.8 g/L NaCl, as described before [70], which previously resulted in the production of N(ε)-acetyl-β-L-lysine. **Fifty milliliters** of exponential-phase biomass was spun down at max speed for 10 min**,** and the supernatant was discarded. The pellet was then resuspended in 1.5 ml of ice-cold acetonitrile:methanol:deionized water (40:40:20) and incubated on ice **for** 30 min. Samples were later centrifuged for 10 min at 4°C and 20 000x*g*, so as to recover the supernatant and remove cell debris. Isomer N(ε)-acetyl-L-lysine (Sigma-Aldrich, St L**o**uis, USA) was dissolved in acetonitrile:methanol:deionized water (40:40:20) at a concentration of 40 μM to serve as negative control. All samples that were employed for the MS2 spectra analysis were diluted 100-fold, including the accumulating metabolite in “***Ca.*** Methanoperedens**.**”

### Fluorescence *in situ* hybridization and polyhydroxyalkanoate staining for confocal laser scanning microscopy

To visualize “*Ca.* Methanoperedens” and the bacterial community, biomass was fixed at three salinities (0%,1.5%, and 3%). Two milliliters of bioreactor samples were fixed with 3% (v/v) paraformaldehyde (PFA) to later perform Double Labeling of Oligonucleotide Probes (DOPE)-FISH for improved signal. Two *“Ca.* Methanoperedens”-specific probes and the EUBmix probe set were employed (Supplementary Table 5) (Biolegio, Nijmegen, The Netherlands). Fixation was followed by sample stabilization using 1% of gelatin with 0.1 g/L chromium sulfate and followed by an overnight hybridization at 46°C with a 35% formamide hybridization buffer, washed (10 min at 48°C) with a washing buffer, ice-cold water-dipped, and air-dried. We checked for autofluorescence with controls that included no probes. Prior to microscopy, samples were embedded in Vectashield with DAPI (Vector Laboratories Inc., Burlingame, USA). Microscopy was performed in a Leica SP8-White Light Laser Confocal Microscope (Leica Microsystems, Wetzar, Germany). For visualizing PHA storage, the same fixed biomass hybridization protocol was combined with Nile Red (10 μl for 10 min) staining (Sigma-Aldrich, Saint Louis, USA) and DAPI-free Vectashield (Vector Laboratories Inc., Burlingame, USA). The FISH probe employed was single-labeled with the Alexa350-fluorophore targeting “*Ca.* Methanoperedens” (NDAMOARCH_641) (Supplementary Table 5), a fluorophore that does not overlap with the Nile Red excitation/emission spectrum (Biolegio, Nijmegen, The Netherlands). We discarded autofluorescence with controls that included no probe or no Nile Red. Confocal microscopy images reported for this study are available at the Image Data Resource FigShare with reference: https://figshare.com/s/1f0f21a456665259077c

### Polyhydroxyalkanoate quantification

Our initial approach involved linking PHA production to *“Ca.* Methanoperedens” by employing Nile Red staining microscopy with *“Ca.* Methanoperedens”-directed FISH. Subsequently, we proceeded to evaluate PHA abundance in the *“Ca.* Methanoperedens” biomass, sampling at salinities 0%, 1.5%, and 3%. Here, PHAs were derivatized to polyhydroxy acids so that the methyl derivative could be analyzed by GC-MS. For that, the freeze-dried samples (10–20 mg) were first redried for 15 min at 100°C in 12 ml crystal vials (sealed with teflon-coated caps). Later, ground dried biomass was extracted with 2 ml of 10% sulfuric acid in methanol with 200 ppm sodium benzoate as internal standard (IS) and 2 ml chloroform. After retightening the caps, samples were heated at 100°C for 20 h. After cooling down for 3 h, samples were vortexed for 1 min with addition of 1 ml milli-Q water and settled for 1 h. From the lower layer (chloroform layer), 1 ml was transferred to a 1.5 ml GC vial.

GC-MS analysis was performed on an Agilent 7890A GC (Agilent Technologies, Santa Clara, CA, USA) equipped with a HP-5MS column (30 m × 0.25 mm × 0.25 μm) using an 7693A automated sampler. The GC was connected to a JEOL JMS-T100 GCv mass spectrometer (JEOL Ltd., Akishima, Tokyo, Japan). For the analysis, 1 μl of each sample was injected (injector temperature 250°C) onto the GC column using a split ratio of 25:1 and the following temperature program: 70°C for 3 min, ramp 10°C/min to 320°C, hold 5 min. A column flow with helium (5.0) of 1.0 ml/min was used. Electron ionization spectra were acquired at 3 Hz (spectra per second) mass range 35–650, column bleeding (silanol 207) in the range 14.50–14.90 min was employed to do internal mass drift compensation for the perfluorokerosene (PFK)-calibrated MS resulting in a mass accuracy window smaller than 3 mmu for mass peaks with sufficient signal to noise. GC-MS peaks were manually integrated using MassCenter (JEOL Ltd., Akishima, Tokyo, Japan). Acquisition was performed using Mass Center System version 2.5.1a © 2001–2010 JEOL Ltd. Spectra were compared to the NIST/EPA/NIH Mass Spectral Library version NIST MS Search 2.3. build May 2017 with the NIST Mass Spectral Search Program.

### Identification and analysis of sialic acids (nonulosonic acids) by mass spectrometry and sialidase activity

The identification and analysis of nonulosonic acids from freeze-dried biomass was performed using acid hydrolysis and DMB labeling, followed by reversed-phase chromatography coupled to high-resolution mass spectrometry PRM scanning using small mass channels as described before [31]. In short, lyophilized biomass was hydrolyzed by 2 M acetic acid for 2 h at 80°C and dried with a Speed Vac concentrator. The released NulOs were labeled using DMB (1,2-diamino-4,5-methylenedioxybenzene dihydrochloride) for 2.5 h at 55°C and analyzed by reversed-phase chromatography Orbitrap mass spectrometry (QE plus Orbitrap, ThermoFisher Scientific, Bleiswijk, Netherlands). For the sialidase activity measurements, lyophilized biomass was added into enough volume of PBS for 60 min to make the biomass swell. Afterward, the samples were centrifuged at 2000 g for 10 min at 4°C. Ten milligrams of the pellet was collected and added into 1 ml of PBS. After vortexing for 1 min, the sialidase activity of the samples was measured. Ten microliters of the homogenized sample was mixed with 40 μl of PBS and 50 μl of 100 μM 4MU-Neu5Ac (2′-(4-methylumbelliferyl)-α-D-N-acetylneuraminic acid) and incubated at 37°C for 60 min. After the addition of 100 μl of 0.5 M sodium carbonate buffer, the fluorescent intensity was measured (ex/em, 355 nm/460 nm) with using 96-well black plates. Ten microliters of PBS was applied as negative control, whereas 10 μl of 0.63 μg/ml AUSA was the positive control. The autofluorescence intensity of each sample was also determined by adding 10 μl of the sample into 90 μl of PBS. The sialidase activity was calculated by subtracting the autofluorescence intensity of the sample from the fluorescence intensity of the sample.

## Results

### “*Ca.* Methanoperedens” partially adapted to increasing salt concentrations

We investigated the biogeography of our “*Ca.* Methanoperedens” species through a database study of publicly available metagenomes. We found that our *Methanoperedenaceae* archaea, “*Ca.* Methanoperedens Vercelli Strain 1,” predominated in permafrost metagenomes (~50% of the total metagenome source recovered), followed by freshwater sediments (~25%), bioreactors, and peat ecosystems (~25%) (Supplementary Fig. 2 and Supplementary Table 2).

**Figure 1:**
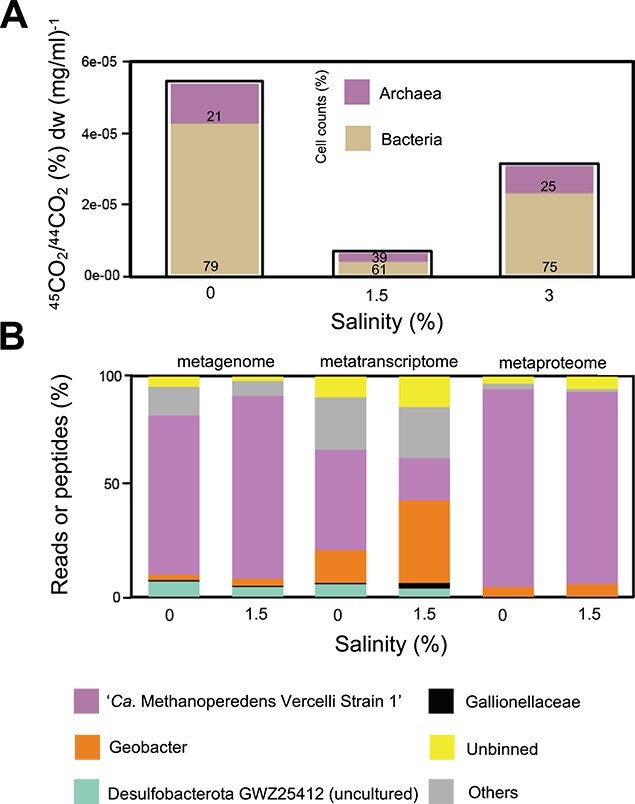
Methane oxidation potential at increasing salinities and meta-omics analysis of the microbial community. (A) Whole bioreactor activity assay with salinity (*x*-axis) and rates from ~4-day cumulative percentage of ^45^C-CO_2_ to ^44^C-CO_2_ ratios in the headspace normalized by biomass dry weight (mg/ml) (*y*-axis). Bar plots are filled with percentages of cell counts of bacteria and archaea, calculated from % 16S rRNA gene DNA copies per μl (purple, archaea; beige, bacteria). The timing for salinities varied: salinity 0%, 1.5%, and 3% were sampled at Weeks 0, 12, and 26, respectively. Salinity 0%–1.5% increase was stepwise with a duration of 12 weeks (0.25% per 2 weeks). Later, the bioreactor ran for 8 weeks at 1.5% salinity. Salinity 1.5%–3% increase was also stepwise but quicker with a duration of 4 weeks (0.5% per week). (B) From left to right: Metagenome bars refer to percentage of mapped reads of a certain MAG; metatranscriptome refers to TPM and metaproteome to relative abundance of top three peptides recovered. Only top population MAGs (>1% mapped reads) are plotted.

To get a first impression on the salt stress response of “*Ca.* Methanoperedens,” we performed microcosm experiments with biomass of a “*Ca.* Methanoperedens” enrichment culture containing “*Ca.* Methanoperedens” originating from a eutrophic freshwater rice paddy field in Italy. With respect to freshwater conditions, direct exposure to 0.5% salinity resulted in a loss of almost half of the activity at the final time point and a total loss of activity at 2% salinity (Supplementary Fig. 3). We still observed methane oxidation at 1%, although with a slower acclimation (Supplementary Fig. 3). To test if gradual exposure to salt stress would result in adaptation, we opted to slowly adapt the “*Ca.* Methanoperedens”-enriched biomass until matching sea water conditions (3%) in a laboratory-scale sequencing batch bioreactor over the duration of ~12 weeks. This led to a methane oxidation activity retention at 1.5% and 3% salinities, whereas the archaea to bacteria cell percentages remained stable (with dry weights of: 1.58, 3.02, and 0.38 mg/μL at salinities 0%, 1.5%, and 3%, respectively) (Fig. 1A).

### Multiomics analysis reveals retention and adaptation of “*Ca.* Methanoperedens” in the microbial community at increased salinities

Sequences affiliated to “*Ca.* Methanoperedens” dominated the enrichment culture with adjusted salinities ranging from freshwater to 1.5%, as 75%–80% of the metagenomes data reads mapped to the top recovered MAGs (Fig. 1B and Supplementary Table 6). Sequences associated to the dominant species MAG “*Ca.* Methanoperedens Vercelli Strain 1” retained their dominance at marine salinities (40%–60% relative abundance) (Fig. 1B and Supplementary Fig. 4). Similarly, six archaeal 16S rRNA gene ASVs classified as “*Ca.* Methanoperedens”: ASV_1 were the most dominant (~97% of recovered total ASVs), followed by ASV_2 (~ 2%) (Supplementary Table 7). The bacterial 16S rRNA gene ASVs were dominated by *Proteobacteria* and unclassified sequences (Supplementary Fig. 5). Bacterial ASVs decreased in abundance and diversity from 0% to 1.5% (Supplementary Fig. 6), with no clear physicochemical abundance-shift patterns (Supplementary Fig. 7).

When assessing metagenome read–based “*Ca.* Methanoperedens” abundance at the three different salinities, we observed congruency with binning results, with read recruitment percentages of 50%, 65%, and 45% at 0%, 1.5%, and 3% salinities, respectively (Supplementary Fig. 4A). “*Ca.* Methanoperedens” captured ~50% of metatranscriptome reads at freshwater salinity, whereas it dropped to below 25% of all TPM at 1.5% (Fig. 1B and Supplementary Table 8). In contrast, the metaproteome indicated that most proteins were mapped to “*Ca.* Methanoperedens” under both 0% and 1.5% salinities with >87% of all proteins assigned to this genus (Fig. 1B and Supplementary Table 9).

### N(ε)-acetyl-β-L-lysine accumulation by “*Ca.* Methanoperedens”

**Figure 2:**
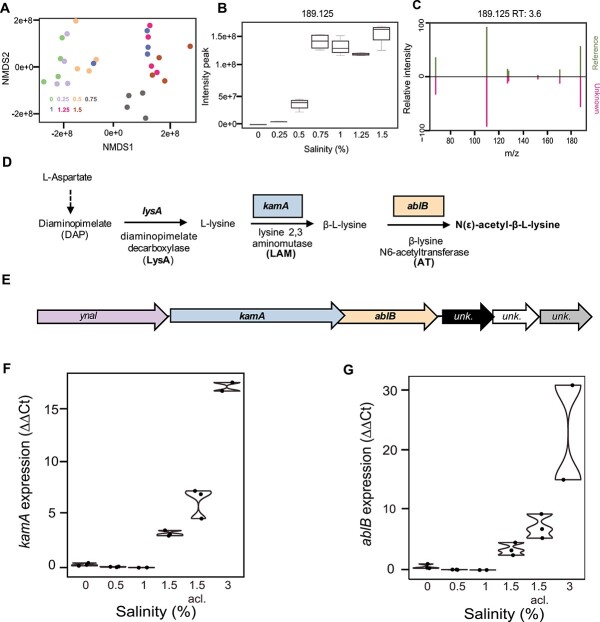
N(ε)-acetyl-β-L-lysine and its biosynthetic gene transcripts increase upon salt stress. (A) NMDS plot of metabolomics samples collected at seven different salinities (in %): 0, 0.25, 0.5, 0.75, 1, 1.25, and 1.5 (*n =* 4 per salinity). Each salinity is depicted using a different color (see legend). (B) Box-and-whisker plot of m/z 189.125 [M + H]^+^ (*n =* 4 per salinity). (C) Mirror plot with MS2 spectra of m/z 189.125 (±10 ppm) from “*Ca.* Methanoperedens” (unknown) and *M. mazei* (reference) demonstrating a spectral match. (D) Osmolyte N(ε)-acetyl-β-L-lysine is a derivative of the L-lysine amino acid family. Synthesis includes the central metabolism core gene encoding diaminopimelate decarboxylase (enzyme LysA encoded by *lysA*) (KEGG: K01586, EC: 4.1.1.20), followed by lysine 2,3-aminomutase (enzyme LAM encoded by *kamA*) (KEGG: K01843, EC: 5.4.3.2) and a β-lysine N6-acetyl transferase (AT encoded by *ablB*) (KEGG: K21935, EC: 2.3.1.264) exclusively found in the “*Ca.* Methanoperedens” MAG in the present enrichment culture studied. (E) Operon gene organization of *kamA* (1326 bp)*-ablB* (870 bp). *kamA-ablB* are preceded (with 1 bp of difference) by the y*naI* gene (1098 bp) (KEGG: K16052), encoding an enzyme similar to a small conductance mechanosensitive channel (MscS) (PFAM: IPR045042) and downstream (41 bp difference) by genes of unknown function. (F, G) Normalized gene expression of “*Ca.* Methanoperedens”-specific *kamA* and *ablB* genes in the form of Δ ΔCt at six different salinities (in %): 0, 0.5, 1, 1.5, 1.5-acclimated, and 3 at weeks 0, 4, 8, 12, 22, and 26, respectively, determined by quantitative PCR (in two to three biological replicates per condition, *n =* 2–3).

To understand the metabolic response to salt stress of “*Ca.* Methanoperedens,” we performed nontargeted LC-MS metabolomics to search for compatible solutes accumulating at seven different salinities from 0% to 1.5%. Overall, we observed that metabolic profiles grouped samples from the range 0% to 0.5% and 0.75% to 1.5%, with distinct groupings in a nonmetric multidimensional scaling (NMDS)-1 plot (Fig. 2A). To filter for osmolytes, we selected LC-MS features that increased to high intensities at higher salinities. This search revealed a top osmolyte candidate with an m/z of 189.125 [M + H]^+^. This feature ranked among the sixth most abundant peak areas out of the 34 330 features detected and was the highest intensity feature that changed with increased salinity (Fig. 2B). *In silico* structure prediction based on the tandem mass spectrometry (MS2) fragmentation of m/z 189.125 yielded as likely identity the known osmolyte N(ε)-acetyl-β-L-lysine. To validate this prediction, we grew the methanogen *M. mazei* under the same salinity (23.8 g/L) that was previously reported to produce N(ε)-acetyl-β-L-lysine [70]. The retention time (RT: 3.6 min) and MS2 fragmentation spectrum of N(ε)-acetyl-β-L-lysine produced by *M. mazei* matched those of the “*Ca.* Methanoperedens” candidate osmolyte (Fig. 2C and Supplementary Fig. 8). To rule out the possibility that the accumulating metabolite was not N(ε)-acetyl-β-L-lysine but its common isomer (N(ε)-acetyl-L-lysine), we performed MS2 experiments of the isomer as “negative control” and confirmed that the MS2 spectra did not match (Supplementary Fig. 8). To conclude, we confirmed that “*Ca.* Methanoperedens” produced the compatible solute N(ε)-acetyl-β-L-lysine.

### N(ε)-acetyl-β-L-lysine production pathway and biosynthetic operon organization

N(ε)-acetyl-β-L-lysine is synthesized from L-lysine (Fig. 2D). N(ε)-acetyl-β-L-lysine requires two enzymes for its synthesis: lysine-2,3-aminomutase (LAM, encoded by *kamA*) and β-lysine-N^6^-acetyl transferase (AT: encoded by *ablB*) (Fig. 2D). In our enrichment culture, the only *ablB* gene in the metagenome data was linked to the dominant “*Ca.* Methanoperedens Vercelli Strain 1” MAG, suggesting that this strain is responsible for its production (Supplementary Table 10). The *kamA* and *ablB* genes were present in the same operon together with an MscS-like *ynaI* gene, potentially encoding a small conductance mechanosensitive channel-like membrane protein (Fig. 2E).

To accurately quantify *kamA* and *ablB* mRNA expression levels, we developed a targeted approach via reverse transcriptase (RT)-qPCR. A low baseline expression of both these genes was measured up to and including 1.5% salinity (Fig. 2F and G). Thereafter, the expression increased stepwise and was highest in the 3% salt conditions (Fig. 2F and G and Supplementary Fig. 9). These observations aligned with the microcosm experiments where “*Ca.* Methanoperedens” was unable to cope with a sudden salinity increase but was able to tolerate the increase when longer incubated allowing for the upregulation and production of the osmolyte N(ε)-acetyl-β-L-lysine (Supplementary Fig. 3).

### Phylogenetic distribution suggests horizontal transfer of genes encoding enzymes for N(ε)-acetyl-β-L-lysine synthesis

**Figure 3:**
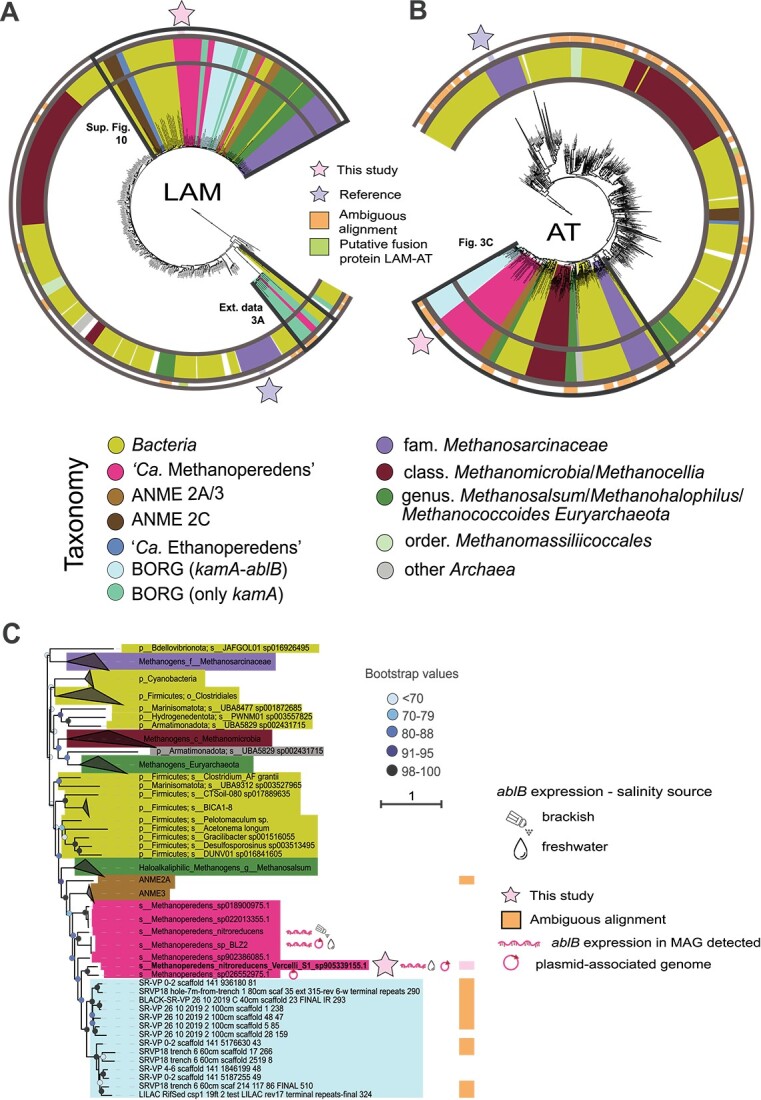
Lysine 2,3-aminomutase (LAM) and β-lysine N6-acetyl transferase (AT) amino acid tree. (A) Taxonomical color-coded overview of LAM and (B) AT gene tree with a particular focus on ANME, methanogens, and BORG-encoded sequences. Putative fusion proteins refer to contigs containing double-annotated *kamA* and *ablb* genes whereas ambiguous alignment indicate sequences that did not surpass the homogeneity character composition established by Iqtree. (C) Zoomed-in view on “*Ca.* Methanoperedens” neighboring sequences in the AT tree in (B) (indicated with black lines). Bootstrap support values for 1000 replicates analysis. Branch length depict average number of amino acid substitutions per site. See Supplementary Fig. 10 for a zoomed-in version of “*Ca.* Methanoperedens” neighboring sequences in LAM tree [black lines in (A)]. Sequences were assigned to their lowest GTBD-Tk v2.1.0 taxonomical category or clustered in clades and refer to their higher taxonomical categories or groups. BORG labels were remained unchanged from original publication. “*Ca.* Methanoperedens” *ablB* gene expression was classified depending on the salinity source employed on the different metatranscriptomic datasets. Annotated sequences included previously observed AT-encoding gene *ablB* expression (obtained from public metatranscriptomes) or *ablB*-harboring “*Ca.* Methanoperedens” that are known to harbor a non-*ablB* carrying plasmid.

To learn more about the phylogenetic distribution of N(ε)-acetyl-β-L-lysine production in ANME archaea, we retrieved KEGG-annotated *kamA* and *ablB* genes, which encode the LAM and AT enzymes, respectively, from all genomes in the GTDB. For the “*Ca.* Methanoperedens”-associated BORG sequences, 50 *kamA* and 37 *ablB* gene-encoding amino acid sequences were manually retrieved from the original publication [35]. Both genes, *kamA* and *ablB*, were categorized according to their taxonomy, with a particular focus on archaeal subgroups (Fig. 3A and B and Supplementary Tables 3 and 4).

Both *kamA* and *ablB* were widely distributed in bacteria and methane/alkane cycling archaea, both chromosomally and encoded on BORGs. Although most BORGs harbored both genes, a small fraction were only carrying *kamA* (Fig. 3 and Supplementary Fig. 10). The “*Ca.* Methanoperedens” *kamA* and *ablB* from this study (Fig. 3A and B, pink star) clustered phylogenetically with those genes from other ANME archaea and were distantly related to the first reported reference in methanogenic archaea (Fig. 3A and B, blue star). Furthermore, expression of the *kamA* and *ablB* genes closely related to those from our “*Ca.* Methanoperedens” have been reported in metatranscriptomic dataset from both brackish and freshwater origins (Fig. 3C and Supplementary Fig. 10). Both *kamA* and *ablB* genes were identified in MAGs representing the ANME-2a, 2c, and 3, but not the 1 and 2B subgroups. Although *kamA* and *ablB* genes from ANME groups clustered close to each other, the methanogenic *kamA* and *ablB* genes appeared to have a wider distribution that is intertwined with those encoded by bacteria (Fig. 3A and B). Our data suggest potential horizontal gene transfer (HGT) of *kamA* and *ablB* from bacteria to ANME and halophilic methanogen *Methanosalsum*, with the “*Ca.* Methanoperedens” *kamA* and *ablB* genes being closely related to those from the bacterial phylum *Firmicutes* (Fig. 3C for *ablB* and Supplementary Fig. 10 for *kamA*).

### Metatranscriptome and metaproteome changes during salt stress

**Figure 4:**
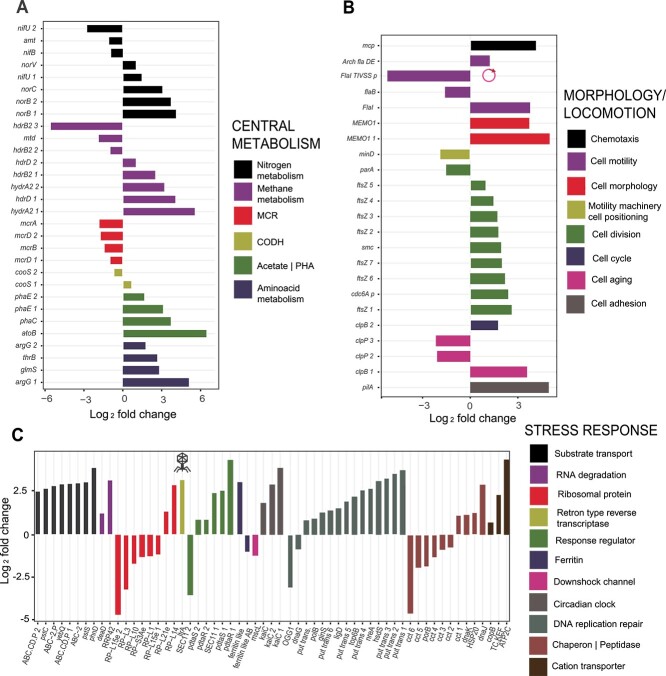
Three-way categorization of top differentially expressed genes (*P* < .05) of “*Ca.* Methanoperedens” between 0% and 1.5% salinities. Categories include central metabolism (A), morphological change and locomotion (B), and overall stress-related response (C). Up- and downregulated transcripts are indicated in the *y*-axis for (A) and (B) and *x*-axis for (C). Up- and downregulated genes are referred to as log_2_ fold change. For (B) and (C), additional annotation includes a plasmid-harboring and potential phage-related transcript, *FlaI_TIVSS* and *ltrA*, respectively.

In addition to the transcripts of *kamA* and *ablB* genes, a total of 286 “*Ca.* Methanoperedens” transcripts were upregulated at nonacclimated 1.5% salinity as compared to freshwater conditions, accompanied by 11 proteins being more abundantly present. Conversely, 264 “*Ca.* Methanoperedens” transcripts were downregulated, and 17 proteins were less abundantly present (threshold: *P* < .05 difference between the 1.5% and 0% salinity) (Fig. 4 and Supplementary Fig. 11). Overall, the metaproteome seemed to exhibit a delayed response to the salt stress. Not only the amount of significantly shifted proteins was notably fewer than transcripts, but the majority of the proteins remained assigned to “*Ca.* Methanoperedens” after 12 weeks of salinity increase (Fig. 1B and Supplementary Table 9).

We subdivided all differentially expressed transcripts into three different categories: central metabolism, cell morphology/locomotion, and overall stress-related response. The methyl-coenzyme M reductase complex (MCR) genes had a lower relative expression at increased salinity (Fig. 4A). Besides, genes linked to nitrosative stress response (nitric oxide reductases *norB* and *norC*) increased in expression (Fig. 4A), in accordance with the accumulated nitrite and reduced ammonium production observed prior to sampling (Supplementary Fig. 1A). Transcripts encoding proteins involved in small amino acid biosynthesis with simpler molecular structure and smaller molecular weight, and other potential compatible solutes, in addition to N(ε)-acetyl-β-L-lysine, were also upregulated (*argG_1*, *argG_2*, and *glmS*; Fig. 4A).

Morphology-associated transcripts were more abundantly expressed: genes encoding for the cell division machinery (*ftsZ*) and flagellar biosynthesis were upregulated (Fig. 4B). However, microscopy imaging was of too-low resolution to visualize “*Ca.* Methanoperedens” morphotype shift from granules to free-living or planktonic cells (Supplementary Fig. 12). We also observed a general increase in stress-related transcripts, with a particular increase in genes encoding ABC-transporters (categorized in substrate transport), putative transposases (categorized as DNA replication repair), and cation transporters (Fig. 4C). Metatranscriptomic data indicated a downregulation of a large mechanosensitive (MS) channel (MScL)-encoding *mscL* (Fig. 4C). Another stress-related transcript was a gene encoding a heme-containing ferritin-like protein (belonging to the cd00657 family) and a nonheme-containing ferritin-like AB (archaea and bacteria) (classified as cd01045 subfamily of ferritins), which were up- and downregulated, respectively (Fig. 4C). We also investigated additional potentially relevant extrachromosomal gene expression shifts upon salt stress. Our same study culture was previously described to lack BORGs, although two plasmids were found [62]. In this study, we managed to retrieve one of the previously well-curated and predominant (highest coverage) plasmids, HMp_v2, from our metagenome (contig id: contigs_NODE_75__2494.086047). This plasmid, composed of 148 open reading frames (ORFs), had nine and one up- and downregulated transcripts, respectively (Supplementary Tables 8 and 10). Among the significantly downregulated genes, we identified a gene encoding a protein involved in cell motility (FlaI_TIVSS_p) (Fig. 4B). A gene encoding a retron-type reverse transcriptase (*ltrA*) was upregulated, suggesting a prophage-related shift (Fig. 4C).

We paired the metatranscriptomic observations to the metaproteome. Some proteins of interest with yet uncharacterized function included a putative spore-production linked protein (YtfJ), which was significantly downregulated (Supplementary Fig. 11). Additionally, a cell-division-related protein belonging to the “SepF_superfamily” was significantly upregulated from 0% to 1.5% salinity (Supplementary Fig. 11).

### PHA in “*Ca.* Methanoperedens” decreased at increasing salinities

**Figure 5:**
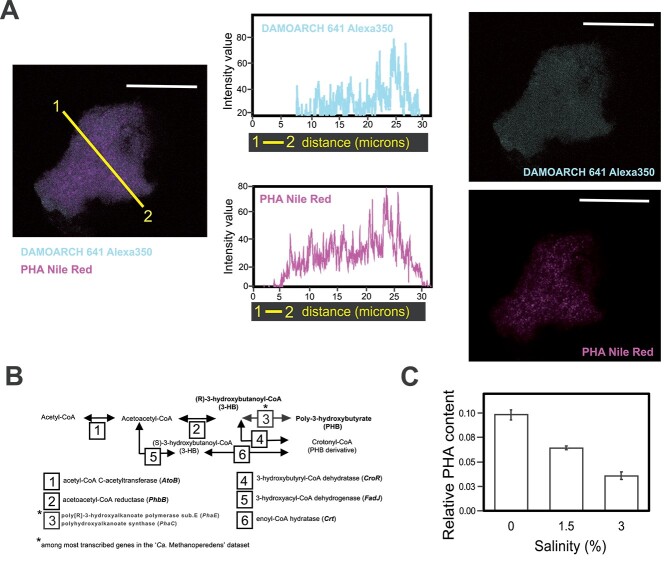
Linking “*Ca.* Methanoperendes” to PHA production and decrease upon salt stress. (A) From left to right: 1.5% salinity “*Ca.* Methanoperedens” biomass labeled with Alexa350-fluorophore-labeled DAMO-ARCH-641 probe signal coupled to Red Nile (PHA stain) signal. Intensity plots from color-coded cross section (yellow line) displaying the Alexa350 signal (blue) and Nile Red signal (pink). Separate imaging from the overlapped signals of Alexa350-fluorophore-labeled N-DAMO-ARCH 641 and Nile Red for the same image. The micrographs are representatives of two independent experiments with two to five granules per well (*n =* 3) at two salinities (0% and 1.5%) per experiment. See Supplementary Fig. 6 for additional micrographs. All scale bars represent 20 μm. (B) Reconstruction of the poly-3-hydroxybutyrate (PHB) and crotonyl-CoA (PHB derivative) production pathways, highlighting the top abundant transcript found on the “*Ca.* Methanoperedens” metatranscriptomes for the studied culture. Genes indicated with an asterisk refer to transcripts with top TPM (relative abundance) within the “*Ca.* Methanoperedens” total transcripts. (C) Quantification/ratio results of the summed area peaks of PHB and crotonyl-CoA identified by GC–MS, normalized with an IS and dry weight (in milligrams). Relative PHA content refers to PHA derivatives area normalized by internal standard and weight (milligrams).

“*Ca.* Methanoperedens” stained positive for PHA accumulation in our enrichment culture at salinities 0% and 1.5% (Fig. 5A, Supplementary Fig. 13). This observation aligned with the high expression of genes encoding PHA synthase in the metatranscriptome data (*pha*C and *pha*E; top 3% of genes of the total “*Ca.* Methanoperedens” expression at 1.5%; Fig. 5B, Supplementary Tables 8 and 10). We also quantified PHA derivatives at 0%, 1.5%, and 3% salinities and observed a clear decrease per biomass weight (Fig. 5C). Albeit the metatranscriptome indicated a significant upregulation of the *phaE* and *phaC* subunits (Fig. 4A), we observed a decrease in abundance of the PhaE enzyme at 1.5% salinity in the proteome dataset (Supplementary Fig. 11).

### Sialic acids (nonulosonic acids) are present in the “*Ca.* Methanoperedens” enrichment

**Figure 6:**
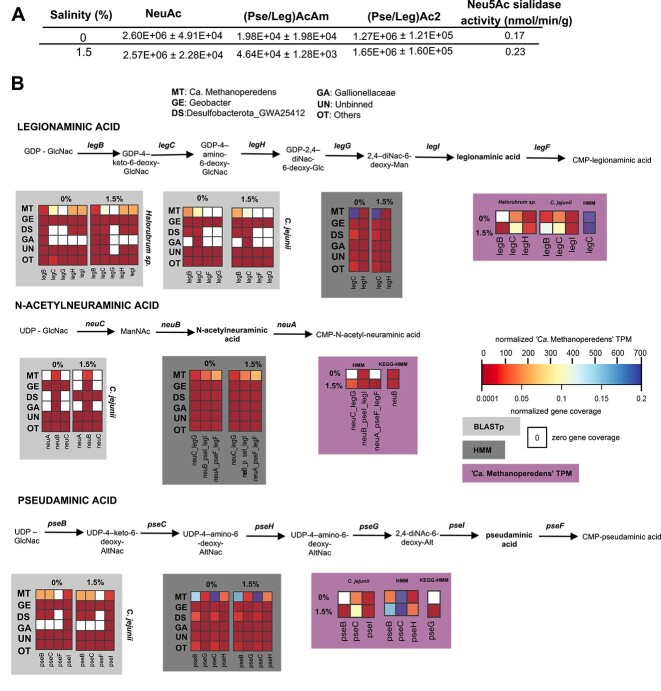
Measured sialic acids/sialidases and reconstructed putative sialic acid production pathways in “*Ca.* Methanoperedens” enrichment. (A) Mass spectrometry peak areas of identified sialic acids (nonulosonic acids, NulOs) in both samples (with intensity peak relative to KDN or deaminated neuraminic acid/standard) including sialidase activity. 5-N-acetylneuraminic acid [Neu(5)Ac], pseudaminic acid (Pse), and legionaminic acid (leg). Pse and Leg modifications are indicated as Leg-AcAm(acetamidino) and Leg-Ac2(acetyl). Pse and Leg have the same molecular weight and thus cannot be differentiated. (B) Metagenomic-based metabolic reconstruction of the production pathways of the three most common NulOs and from which abundant peaks with matching accurate mass could be obtained: NeuAc and Pse/Leg with their respective modifications. Heatmaps refer to “*Ca.* Methanoperedens” and bacterial side-community-normalized gene coverage queried via BLASTp (light gray) or HMM profiles (dark gray). Some BLASTp (against *Halorubrum* sp. PV6 & *Campylobacter jejunii*) and HMM sequence hits overlapped. Heatmaps were also employed to depict metatranscriptomic-based normalized gene expression profiles (TPM) calculated only considering “*Ca.* Methanoperedens” transcripts expressed under 0% and 1.5% salinities (pink). Heatmap legend to the middle right of the panel refers to normalized “*Ca.* Methanoperedens” TPM (from 0 to 700), whereas the one below depicts metagenomic-based overall community normalized gene coverage (0.0001–0.2).

Our study furthermore aimed to expand the knowledge on sialic acids (nonulosonic acids) as a potential mechanism of adaptation to salinity stress for “*Ca.* Methanoperedens.” There was a marked difference in the abundance of pseudaminic acid/legionaminic acid–type nonulosonic acids—measured as (Pse/Leg)AcAm and (Pse/Leg)Ac2)—but not for *N*-acetylneuraminate (Neu(5)Ac)-type nonulosonic acid (Fig. 6A). Moreover, we observed changes in sialidase activity for a Neu(5)Ac probe (Fig. 6A). We then investigated the metabolic potential for sialic acid production by metagenomics. “*Ca.* Methanoperedens” harbored the potential for sugar conversions of the measured sialic acids (Fig. 6B). *Geobacter* seemed to be the most versatile community member, as it appeared to be able to independently produce the three different types of sialic acids measured (Fig. 6B). “*Ca.* Methanoperedens” still contributed the most to the overall gene coverage, considering that it dominated the enrichment both at 0% and 1.5% salinities. Furthermore, we retrieved low transcription levels for some genes encoding sialic acid production enzymes of “*Ca.* Methanoperedens,” for both freshwater and brackish salinities (Fig. 6B). For those transcripts, a low general increase in expression could be observed (Fig. 6B).

## Discussion

Our present study demonstrates that “*Ca.* Methanoperedens” acclimates to marine salinities (3%) and synthesizes N(ε)-acetyl-β-L-lysine during 12 weeks of salt increase (from 0% to 1.5%). This finding represents the description of a relevant osmolyte in the ANME group, achieved through an untargeted metabolomics approach. Our study demonstrates the power of untargeted metabolomics to discover uncharted metabolites in archaea. This osmolyte was first described in the methanogenic archaeon *M. mazei* [71], although its encoding genes, *kamA* and *ablB*, were later discovered on the chromosomes of several methanogenic archaea [70]. N(ε)-acetyl-β-L-lysine was also found in halophilic bacteria [72, 73] and was reported as a key excretion osmolyte upon hyposalinity stress in anaerobic granular sludge-associated microbial consortia [74].

Gene organization analysis of the N(ε)-acetyl-β-L-lysine key genes *kamA* and *ablB* encoding for its biosynthetic pathway shows that they are encoded in the same operon with an additional gene encoding for a poorly described medium-sized small conductance mechanosensitive channel (MscS)-like membrane protein: *ynaI* [75]. So far, the homologous MscS protein from *Escherichia coli* is the best characterized mechanosensitive channel from this protein family and it protects *E. coli* during hypoosmotic shock [76–78]. Although there is no direct physiological evidence on the role of YnaI yet [79], our data indicate an upregulation of *ynaI* under increased salinities.

Our metagenome indicated that *ablB* is only found in “*Ca.* Methanoperedens” in the here-investigated culture and thus directly linking N(ε)-acetyl-β-L-lysine to ANME in the enrichment. Only low expression of *kamA* and *ablB* was found in the metatranscriptome, possibly due to (too) late sampling relative to the salinity increase. Dedicated RT-qPCR experiments with “*Ca.* Methanoperedens”-specific *kamA* and *ablB* primers at six different time points and salinity levels revealed strong salinity-dependent upregulation of this gene cluster. In addition, we decided to screen for evidence of *ablB* expression in publicly available *“Ca.* Methanoperedens” metatranscriptomes. For the freshwater metatranscriptomes, the expression levels (measured as TPM) were very low, with values ranging from ∼0 to 2 TPM [80] to ∼0 to 1 TPM [62] (Fig. 3C and Supplementary Fig. 10). For the brackish “*Ca.* Methanoperedens” metatranscriptomes, values remained low, with ∼80 TPM (planktonic) and ∼40 TPM (granular) [27] (Fig. 3C and Supplementary Fig. 10).

The “*Ca.* Methanoperedens” LAM (encoded by *kamA*) and AT (encoded by *ablB*, Fig. 3A and B) cluster phylogenetically with those encoded by other ANME archaea and are distantly related to the first reported reference in methanogenic archaea [70] (Fig. 3A and B). No LAM or AT were retrieved from ANME1 and ANME2B groups, suggesting either limited genome coverage of these lineages or their use of different physiological strategies to cope with salinity stress.

Several *kamA* and *ablB* genes were encoded on extrachromosomal BORG elements, as reported in the original BORG study [35]. The nature of these elements and gene assimilation process from associated chromosomes is still a conundrum. Al Shayeb *et al.* claimed that they found not enough evidence to prove or discard them as archaeal viruses, plasmids, or minichromosomes. Finally, these elements were classified as novel extrachromosomal elements closest probably to megaplasmids, which, so far, have only been reported associated with “*Ca.* Methanoperedens.” In their study, “*Ca.* Methanoperedens” was also found to encode key functional enzymes such as the MCR complex and genes associated with stress response systems, including putative N(ε)-acetyl-β-L-lysine production potential [35]. Taking all together, the presence of N(ε)-acetyl-β-L-lysine biosynthesis genes in both “*Ca.* Methanoperedens” chromosome and BORGs makes *kamA* and *ablB* suitable candidates for phylogenetic studies. On the one hand, we showed that BORGs might have assimilated the LAM and AT encoding *kamA* and *ablB*-gene operon from a close relative of our current study’s “*Ca.* Methanoperedens Vercelli Strain 1” (Fig. 3C and Supplementary Fig. 10). In addition, our phylogenetic analysis suggested HGT events from bacteria to ANME archaea. In fact, both *kamA* and *ablB* genes from ANME root back to those from Firmicutes species. Although it has been documented that “*Ca.* Methanoperedens” is particularly receptive to the acquisition of genes from a variety of bacteria and archaea [81], the role of the BORG elements in such a transfer is unclear.

An exploratory visualization of upregulated stress-related transcripts pointed out some related to the production of arginine and glutamine (*argG* and *glmS*)*,* which have been well established as compatible solutes [82, 83] (Fig. 4A). Another clear transcriptional shift was the high expression of genes encoding for cation transporters (Fig. 4C). These transporters could be involved in regulating cellular osmotic pressure, as reported for a nitrate-dependent methanotroph culture when exposed to marine salinities [26]. We also observed a different response for two different types of ferritin, classified as “ferritin-like” family (upregulated) and “ferritin-like AB” family gene (downregulated) (Fig. 4C). Although the latter still holds an uncharacterized function, the ferritin-like upregulated transcript, similarly to the bacterioferritin, has been linked to a range of functions such as iron regulation and reactive radical production [84]. We also observed the upregulation of a gene encoding for a large mechanosensitive channel, *mscL*, which helps cells cope with hypoosmotic conditions [76]. This is in agreement with the observed downregulation of *mscL* upon hyperosmotic stress (Fig. 4C).

Our study indicates a putative salt-stress trigger of a morphology shift as we detected an upregulation of several morphology-associated transcripts (Fig. 4B). Recently, this pleomorphic shift was reported for “*Ca*. Methanoperedens nitroreducens” [27]. Here, granular/PHA-accumulating “*Ca.* Methanoperedens” cells were differentiated from their planktonic free-living rod-shaped morphotypes. Such transcript upregulation was also previously observed in an oxygen-stress “*Ca.* Methanoperedens” study [85]. Similarly, some *Methanosarcina* species have been reported to disaggregate and grow as a single cell upon salt exposure [86]. Taken together, this indicates a potential link between stress and phenotypic shifts. However, considering that our enrichment ran as a sequencing batch reactor (SBR), planktonic cells are likely to be washed out of the system with regular exchange of the supernatant media (Supplementary Fig. 1B). The bioreactor system employed might explain why we observed such low signals of planktonic cells in our micrographs (Supplementary Fig. 12).

The “*Ca.* Methanoperedens” investigated in this study was linked to the diminishment of PHA upon salt exposure (Fig. 5C). These observations align with recent observations on PHA decrease in a nitrate-dependent methane oxidizing enrichment exposed to marine salinities [26]. Furthermore, we described the presence of three different ecologically underexplored sialic acids in our enrichment as well as their putative role upon salt stress. Our “*Ca.* Methanoperedens” MAG encoded the biosynthesis pathway for the full production of one of them, legionaminic acid, which has previously been described in the halophilic archaeon *Halorubrum* sp. PV6 [57] (Fig. 6B).

To conclude, we report the metabolic and physiological response of freshwater anaerobic methanotroph “*Ca.* Methanoperedens Vercelli Strain 1” to salinity increase. Although not primarily found in marine environments (Supplementary Fig. 2 and Supplementary Table 2), we show that “*Ca.* Methanoperedens” could tolerate a wide range of salinities, from freshwater to marine conditions. We identified the production of the key osmolyte N(ε)-acetyl-β-L-lysine and linked it to “*Ca.* Methanoperedens.” Furthermore, we expanded the known phylogenomic distribution of the key biosynthetic *kamA* and *ablB* genes–encoding proteins across an archaeal-focused universal evolutionary tree. We propose that these genes may have been horizontally transferred from the bacterial phylum *Firmicutes* to the ANME archaea and halophilic methanogen *Methanosalsum*.

We further reveal the putative roles of carbon storage polymers (PHAs) and extracellular polysaccharide structures as coping mechanism to salt stress in “*Ca.* Methanoperedens.” Together, this information may help to understand how the anaerobic methane filter in coastal ecosystems will respond to predicted environmental pressures in the future. To further enhance the understanding of natural saline intrusions, the introduction of marine taxa to freshwater microbiomes and resulting competitive pressure should also be addressed. Hence, future research integrating field studies with more complex laboratory experimental designs is needed.

## Supplementary Material

reviewed_w_eic_supplementary_figures_wrae137

rev_SupplementaryTables1_10_wrae137

rev2_SupplementaryFig13_wrae137

## Data Availability

Raw 16S rRNA gene sequences and metagenomics/transcriptomics reads have been uploaded to the European Nucleotide Archive with project number PRJEB70247. Proteomics raw data, reference sequence database, and database search files have been deposited in the ProteomeXchange consortium database with the dataset identifier PXD048239. Microscopic experiments can be accessed via the FigShare project link: https://figshare.com/s/1f0f21a456665259077c
