# Supplementary material for: Osmoregulation in freshwater anaerobic methane-oxidizing archaea under salt stress
Source: ISME J. 2024 Jul 20;18(1):wrae137. doi: 10.1093/ismejo/wrae137 (PMC11337218; doi:10.1093/ismejo/wrae137)
Supplement: reviewed_w_eic_supplementary_figures_wrae137 [file reviewed_w_eic_supplementary_figures_wrae137.docx]

**Supplementary Data for**

**Osmoregulation in freshwater anaerobic methane oxidizing archaea under salt stress**

Maider J. Echeveste Medrano^1^, Andy O. Leu^2^, Martin Pabst^3^, Yuemei Lin^3^, Simon J. Mcllroy^2^, Gene W. Tyson^2^, Jitske van Ede^3^**,** Irene Sánchez-Andrea^4,5^, Mike Jetten^1­­^, Robert Jansen^1^ and Cornelia U. Welte*^1^

***Supplementary Fig. 13** is provided as a separate Supplementary Video and Audio file single separate ppt (16:9) file to secure maximum resolution on Z-stack clips.

****Supplementary Tables 1-6 and 8-10** are provided as a single excel file.

**Supplementary Fig. 1: Physicochemical monitoring of the ‘*Ca.* Methanoperedens’-enrichment culture over the course of the experiment.** **A** Bioreactor liquid nitrogen -nitrate (NO_3_^-^), nitrite (NO_2_^-^) and ammonium (NH_4_^+^)- concentration in mmol. Medium nitrate-feed is indicated as mmol/day. Black dots indicate when whole-bioreactor 1-week activity assays were performed at the three different salinities (in %): 0, 1.5 and 3. **B** Hydraulic retention time (HRT) and base consumption are indicated in days and ml/week, respectively. Note that medium nitrate-feed, nitrite accumulation or 1-week activity assay time spans (panel A) correlate with no HRT or “fed-batch” bioreactor operation-system shift (panel B).

**Supplementary Fig. 2: ‘*Ca*. Methanoperedens Vercelli Strain 1’ read-based biogeography**

Sandpipper-enabled SingleM-search of ‘*Ca.* Methanoperedens Vercelli Strain 1’ taxonomical marker search in the Sequencing Read Archive (SRA). See Material and Methods for SingleM specifics and Table S2 for Sequencing Read Archive (SRA) references per natural or engineered ecosystem.

**Supplementary Fig. 3: Methane oxidation activity assay microcosm experiment at increasing salinity over time.** Tested salinities (black; 0.5% circle, 1% triangle, 2% diamond, 3% square) and controls (gray; dots and dotted line, control without nitrate; solid line and squares, positive control/0% salinity). Note that freshwater and 0.5% salinities were performed in duplicates (n=2) while the rest of the conditions had a single replicate (n=1). Y-axis indicates percentage of ^45^C-CO_2_ to ^44^C-CO_2_ ratios in the headspace. Nitrate additions (2mM) are indicated with arrows (day 0 and 4).

**Supplementary Fig. 4: Metagenomic read and omics-based ‘*Ca.* Methanoperedens’ presence/activity monitoring. (A)** Kaiju-based taxonomical read-classification of three metagenomes collected at 0, 1.5% and 3% salinity. Note that metagenomes at 0% and 1.5% had deeper sequencing than 3%. **(B)** Read binning-capture overview and assembly/binning statistics belonging to 0% and 1.5 salinity metagenomes. Single copy marker ribosomal protein L6 (*rplf*)-read counts of top taxa indicated as Operational Taxonomical Unit (OTU) counts for the two different salinity-metagenomes. From top to bottom, binned vs unbinned reads. **(C)** Overview of assembled and binned read counts (and percentages) mapped to total reads recovered per metagenome

**Supplementary Fig. 5: Overview of bacterial community composing the enrichment between 0% to 1.5% salinities.** Major taxa (x-axis) composing at least 1% or less of the 16S rRNA gene reads recovered are depicted with a black dot. Major taxa composing more than 1% of the reads recovered are indicated with different colors per salinity %: green, 0%; purple, 0.25%; yellow, 0.5%; gray, 0.75%; blue, 1%, pink, 1.25%, orange, 1.5%.

**Supplementary Fig. 6: 16S rRNA gene amplicon Amplicon Sequence Variant (ASV)-based decrease in bacterial diversity and richness indexes.** Chao richness **(A)** and Shannon Diversity (**B)** indexes assigned to bacterial-ASVs recovered per each salinity 16S rRNA gene amplicon dataset

**Supplementary Fig. 7: Influence of physicochemical parameters to bacterial 16S rRNA amplicon sequencing variants (ASVs).** Non Metric Multidimensional Scaling (NMDS) plot grouping ASVs per salinity % sampled. Physicochemical parameters in the matrix include: ammonium concentration measured in the bioreactor (br ammonium), variation on nitrate or base consumption over time (nitrate or base consumption), salinity and hydraulic retention time (HRT).

**Supplementary Fig. 8: Confirmation of m/z 189.125 as osmolyte N(ε)-β-acetyl-L-lysine (A)** Chromatograms of m/z 189.125 (± 10ppm) for three different samples: reference osmolyte-producing *Methanosarcina mazei* positive control (N(ε)-acetyl-β-L-Lysine) (green), unknown ‘*Ca.* Methanoperedens’-accumulating (pink) and negative-control **(**N(ε)-acetyl-L-lysine) (brown). **(B)** MS2 fragmentation spectra of isomers with m/z 189.125 (± 10ppm) eluting at three different retention times (RTs), collected at a collision energy of 20 V.

**Supplementary Fig. 9:** Gene expression of *kamA* and *ablB* genes Panels **(A)** and **(B)** depict normalized x-fold expression of *‘Ca.* Methanoperedens’ specific-*kamA* and *ablB* genes in the form of Δ ΔCt (y-axis) at 6 different salinities (in %): 0,0.5,1,1.5, 1.5- acclimated and 3 at weeks 0,4,8,12,22 and 26, respectively. Expression was determined by quantitative PCR and the experiment was performed with three biological replicates per salinity (n=3)


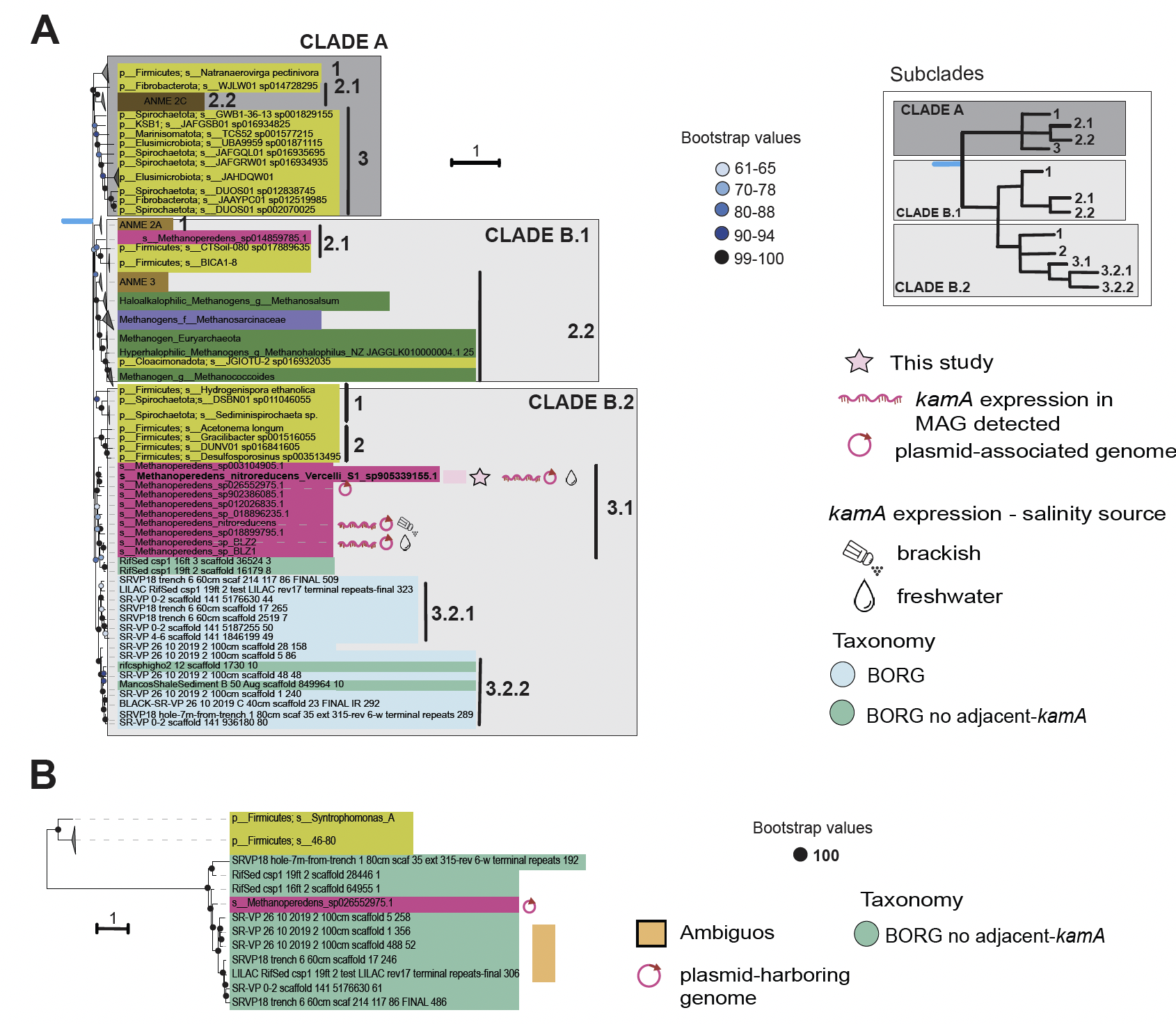


**Supplementary Fig. 10: Detailed lysine 2,3-aminomutase (LAM) tree from Figure 4A.**

**(A)** Close-up from current study’s ‘*Ca*. Methanoperedens’ neighbouring LAM sequences **(B)** Second LAM tree close up from a plasmid-harboring ‘*Ca.* Methanoperedens’ MAG (s_Methanoperedens_sp026552975.1) containing two *kamA* genes in panel A (clade 3.1) and another in panel B with distant relative BORG-associated genes. Sequences were assigned to their lowest GTBD-Tk v2.1.0 taxonomical category or clustered in clades and referred to by their higher taxonomical categories or groups. BORG labels were remained unchanged from original publication. ‘*Ca.* Methanoperedens’ *kamA* gene expression was classified depending on the salinity source employed on the different metatranscriptomic datasets. Ambiguous sequences are those that did not pass the homogeneity character composition established by IQ-TREE. Branch length depict average number of amino acid substitutions per site.

**Supplementary Fig. 11: Top significant differential shifts (*P*<0.05) of ‘*Ca.* Methanoperedens’ proteins between 0% to 1.5% salinities**. Up and downregulated proteins (with well-curated annotations) (x-axis) are expressed as log_2_ fold change (y-axis).

**Supplementary Fig. 12: ‘*Ca.* Methanoperedens’ and bacterial side-community double-labelled probe (DOPE)-FISH imaging at 0%, 1.5% and 3% salinity.** Panels **(A)** and (**B)** correspond to the same Z-stack cut at freshwater and 1.5% salinities, respectively. Panel C and D correspond to the same Z-stack image at 3% salinity at two different depths. For panels **(C)** and **(D)** the image was about (80 by 80) microns in size, with 10 microns imaged-depths. Micrograph C was taken from the lowest depth, next to the slide. This way the planktonic fraction of the granule was imaged. Micrograph D was taken from the highest dept, focused on the granular fraction. From left to right for all panels indicate FLUOS-All Bacteria mixed DOPE-probes (in cyan), overlap of DAMOARCH 641 (pink) and ARCH 915 (yellow) DOPE-probes and all probes mixed together with DAPI signal (dark blue). All salinities depict a consistent ‘*Ca.* Methanoperedens’ to bacterial side community presence. The micrographs are representatives of 2-5 granules per well (n=3 wells per salinity) at three different salinities (0%, 1.5% and 3%) per experiment.

**Supplemental Tables**

**Supplementary Table 7:** Amplicon Sequencing Variants (ASVs) belonging to analyzed archaeal 16S rRNA gene amplicon sequences at seven different salinities from 0% to 1.5%. All ASVs categorized as genus ‘*Ca.* Methanoperedens’.

| **Week** | **0** | **2** | **4** | **6** | **8** | **10** | **12** |
| --- | --- | --- | --- | --- | --- | --- | --- |
| **Salinity (%)** | **0.00** | **0.25** | **0.50** | **0.75** | **1.0** | **1.25** | **1.5** |
| ASV_1 | 109,764 | 99,324 | 93,966 | 99,542 | 93,176 | 112,945 | 81,799 |
| ASV_2 | 2,604 | 2,470 | 2,178 | 2,324 | 2,255 | 2,613 | 1,907 |
| ASV_3 | 161 | 168 | 186 | 236 | 182 | 47 | 51 |
| ASV_4 | 109 | 127 | 121 | 102 | 93 | 107 | 85 |
| ASV_5 | 101 | 0 | 135 | 110 | 142 | 0 | 0 |
| ASV_6 | 0 | 0 | 0 | 0 | 0 | 9 | 0 |

**References Supplementary Data**

Amann, R. I., Binder, B. J., Olson, R. J., Chisholm, S. W., Devereux, R., & Stahl, D. A. (1990, Jun). Combination of 16S rRNA-targeted oligonucleotide probes with flow cytometry for analyzing mixed microbial populations. *Applied and environmental microbiology, 56*(6), 1919-1925.

Caporaso, J. G., Lauber, C. L., Walters, W. A., Berg-Lyons, D., Huntley, J., Fierer, N., Owens, S. M., Betley, J., Fraser, L., Bauer, M., Gormley, N., Gilbert, J. A., Smith, G., & Knight, R. (2012, Aug). Ultra-high-throughput microbial community analysis on the Illumina HiSeq and MiSeq platforms. *Isme j, 6*(8), 1621-1624. <https://doi.org/10.1038/ismej.2012.8>

Daims, H., Bruhl, A., Amann, R., Schleifer, K. H., & Wagner, M. (1999, Sep). The domain-specific probe EUB338 is insufficient for the detection of all Bacteria: development and evaluation of a more comprehensive probe set. *Syst Appl Microbiol, 22*(3), 434-444. <https://doi.org/10.1016/s0723-2020(99)80053-8>

Herlemann, D. P., Labrenz, M., Jürgens, K., Bertilsson, S., Waniek, J. J., & Andersson, A. F. (2011, Oct). Transitions in bacterial communities along the 2000 km salinity gradient of the Baltic Sea. *Isme j, 5*(10), 1571-1579. <https://doi.org/10.1038/ismej.2011.41>

Schubert, C. J., Vazquez, F., Lösekann-Behrens, T., Knittel, K., Tonolla, M., & Boetius, A. (2011). Evidence for anaerobic oxidation of methane in sediments of a freshwater system (Lago di Cadagno). *FEMS Microbiol Ecol, 76*(1), 26-38. <https://doi.org/10.1111/j.1574-6941.2010.01036.x>

Takai, K., & Horikoshi, K. (2000, Nov). Rapid detection and quantification of members of the archaeal community by quantitative PCR using fluorogenic probes. *Applied and environmental microbiology, 66*(11), 5066-5072. <https://doi.org/10.1128/aem.66.11.5066-5072.2000>

Vaksmaa, A., Guerrero-Cruz, S., van Alen, T. A., Cremers, G., Ettwig, K. F., Lüke, C., & Jetten, M. S. M. (2017, Sep). Enrichment of anaerobic nitrate-dependent methanotrophic 'Candidatus Methanoperedens nitroreducens' archaea from an Italian paddy field soil. *Appl Microbiol Biotechnol, 101*(18), 7075-7084. <https://doi.org/10.1007/s00253-017-8416-0>
