# Supplementary material for: Osmoregulation in freshwater anaerobic methane-oxidizing archaea under salt stress
Source: ISME J. 2024 Jul 20;18(1):wrae137. doi: 10.1093/ismejo/wrae137 (PMC11337218; doi:10.1093/ismejo/wrae137)
Supplement: rev2_SupplementaryFig13_wrae137 [file rev2_supplementaryfig13_wrae137.pptx]

## Slide 1
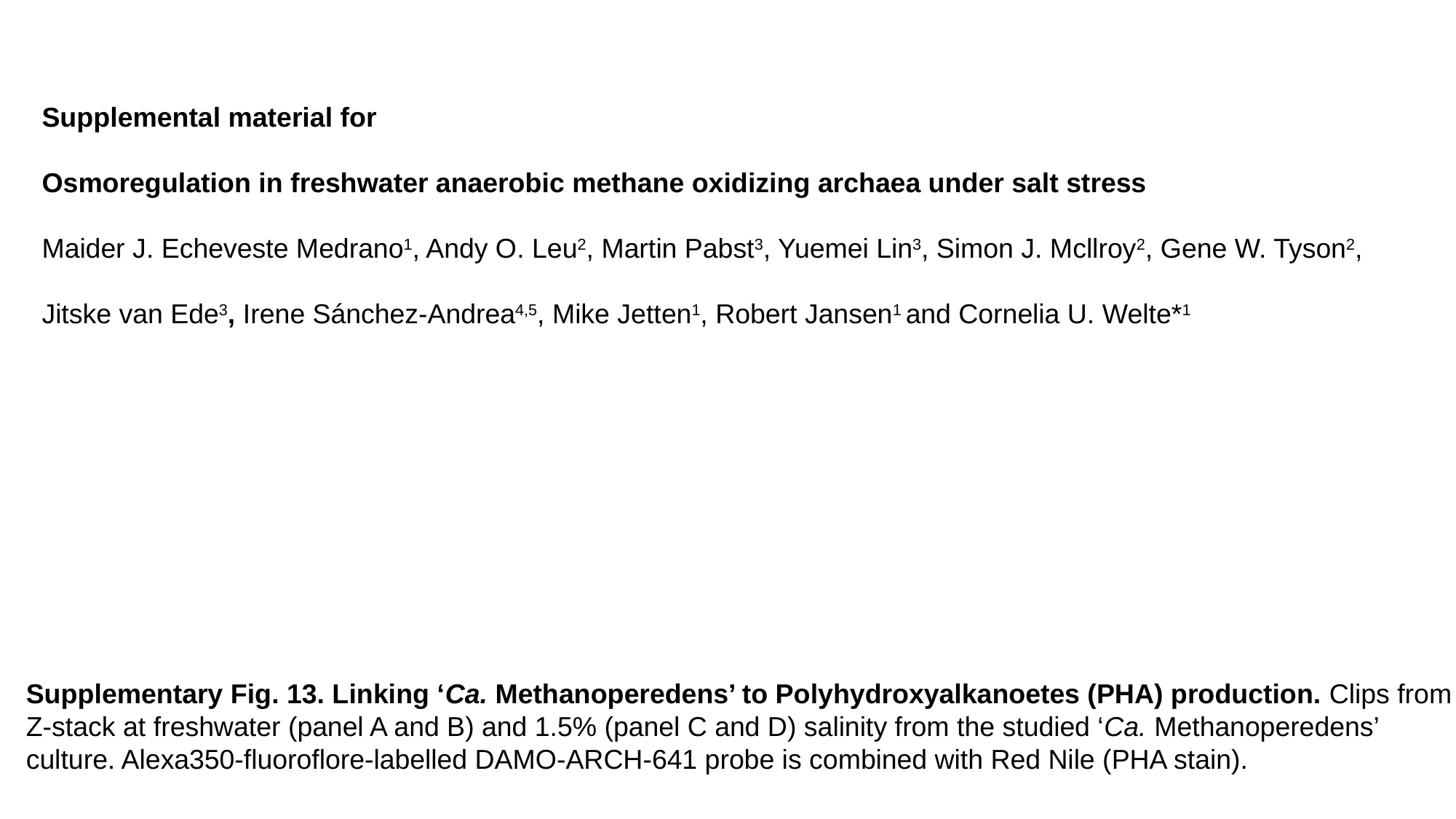

Supplemental material for
Osmoregulation in freshwater anaerobic methane oxidizing archaea under salt stress
Maider J. Echeveste Medrano1, Andy O. Leu2, Martin Pabst3, Yuemei Lin3, Simon J. Mcllroy2, Gene W. Tyson2, Jitske van Ede3, Irene Sánchez-Andrea4,5, Mike Jetten1­­, Robert Jansen1 and Cornelia U. Welte*1
Supplementary Fig. 13. Linking ‘Ca. Methanoperedens’ to Polyhydroxyalkanoetes (PHA) production. Clips from Z-stack at freshwater (panel A and B) and 1.5% (panel C and D) salinity from the studied ‘Ca. Methanoperedens’ culture. Alexa350-fluoroflore-labelled DAMO-ARCH-641 probe is combined with Red Nile (PHA stain).

## Slide 2
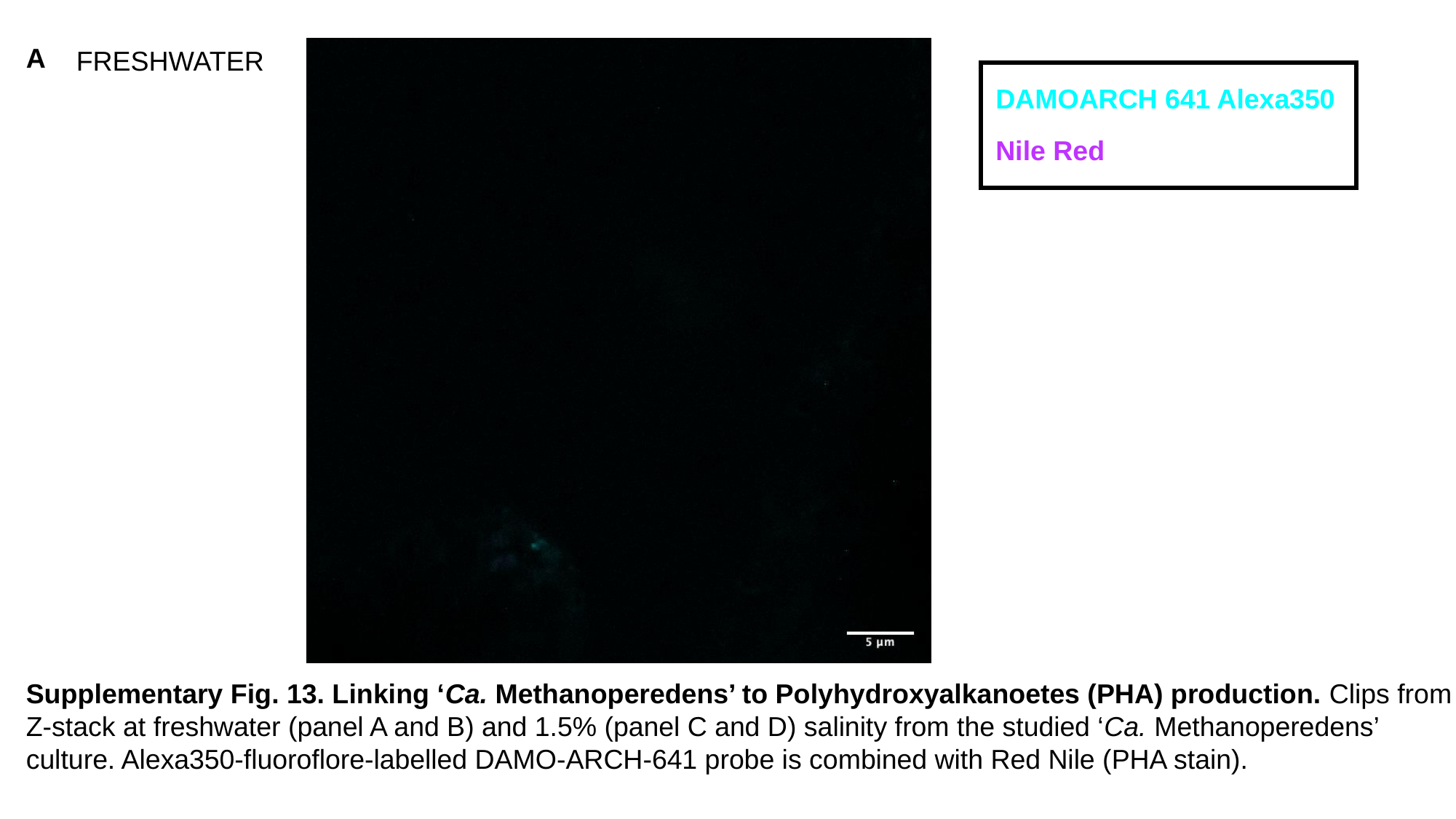

A
FRESHWATER
DAMOARCH 641 Alexa350
Nile Red
Supplementary Fig. 13. Linking ‘Ca. Methanoperedens’ to Polyhydroxyalkanoetes (PHA) production. Clips from Z-stack at freshwater (panel A and B) and 1.5% (panel C and D) salinity from the studied ‘Ca. Methanoperedens’ culture. Alexa350-fluoroflore-labelled DAMO-ARCH-641 probe is combined with Red Nile (PHA stain).

## Slide 3
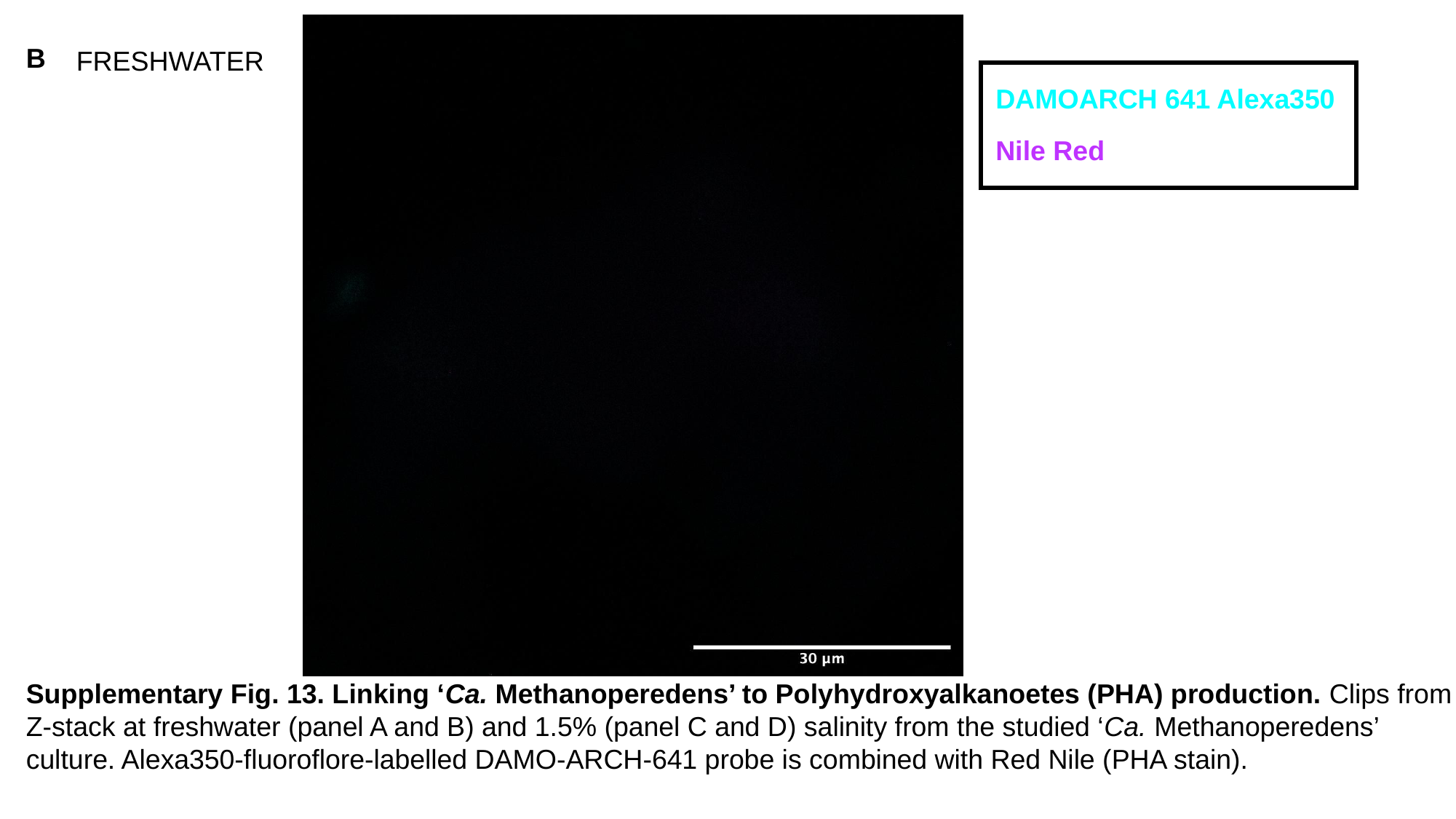

B
FRESHWATER
DAMOARCH 641 Alexa350
Nile Red
Supplementary Fig. 13. Linking ‘Ca. Methanoperedens’ to Polyhydroxyalkanoetes (PHA) production. Clips from Z-stack at freshwater (panel A and B) and 1.5% (panel C and D) salinity from the studied ‘Ca. Methanoperedens’ culture. Alexa350-fluoroflore-labelled DAMO-ARCH-641 probe is combined with Red Nile (PHA stain).

## Slide 4
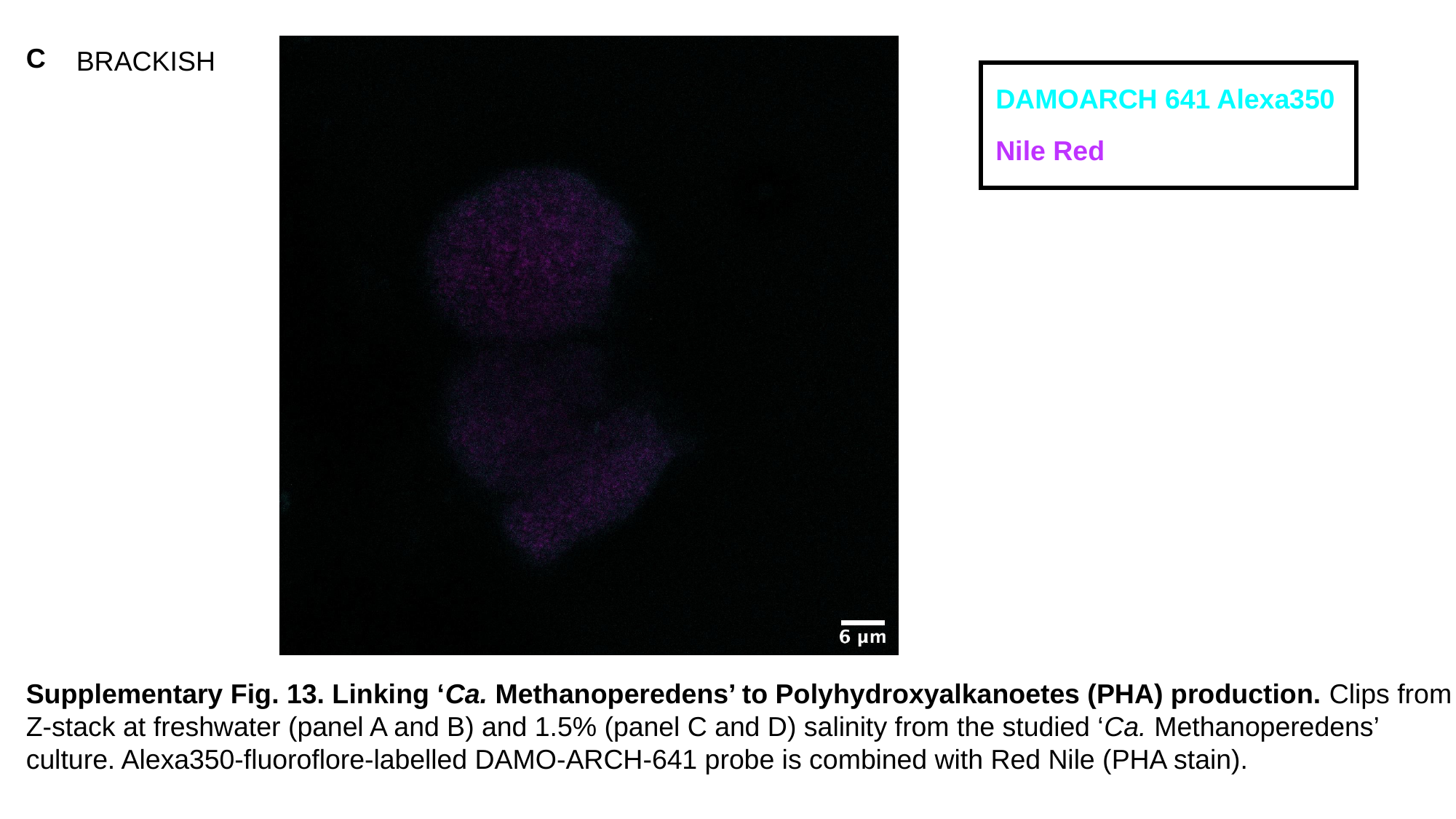

C
BRACKISH
DAMOARCH 641 Alexa350
Nile Red
Supplementary Fig. 13. Linking ‘Ca. Methanoperedens’ to Polyhydroxyalkanoetes (PHA) production. Clips from Z-stack at freshwater (panel A and B) and 1.5% (panel C and D) salinity from the studied ‘Ca. Methanoperedens’ culture. Alexa350-fluoroflore-labelled DAMO-ARCH-641 probe is combined with Red Nile (PHA stain).

## Slide 5
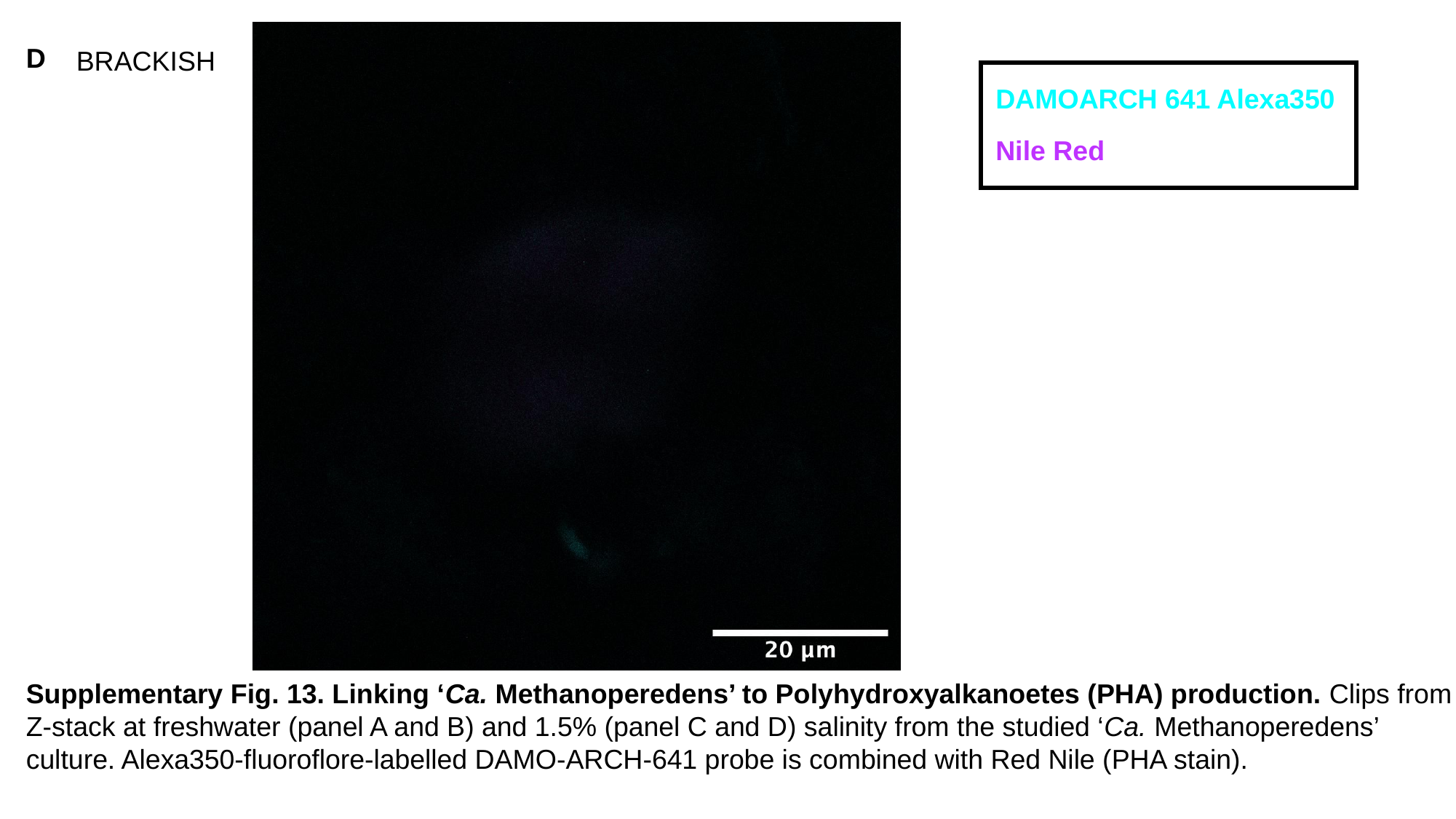

D
BRACKISH
DAMOARCH 641 Alexa350
Nile Red
Supplementary Fig. 13. Linking ‘Ca. Methanoperedens’ to Polyhydroxyalkanoetes (PHA) production. Clips from Z-stack at freshwater (panel A and B) and 1.5% (panel C and D) salinity from the studied ‘Ca. Methanoperedens’ culture. Alexa350-fluoroflore-labelled DAMO-ARCH-641 probe is combined with Red Nile (PHA stain).
